# Development and Psychometric Assessment of a Chinese Version of the Ultra-Low Vision Visual Functioning Questionnaire-50

**DOI:** 10.1167/tvst.13.11.20

**Published:** 2024-11-18

**Authors:** Jing Cong, Xinyuan Wu, Chunqiong Dong, Jing Wang, Chenli Feng, Gechun Wang, Yiting Wu, Gislin Dagnelie, Jinhui Dai, Yuanzhi Yuan

**Affiliations:** 1 Department of Ophthalmology, Zhongshan Hospital, Fudan University, Shanghai, PR China; 2 Department of ophthalmology, hospital of Zhejiang people's armed police, Hangzhou, PR China; 3 Lions Vision Center, Wilmer Eye Institute, Johns Hopkins University School of Medicine, Baltimore, MD, USA; 4 Zhongshan Hospital (Xiamen), Fudan University, Xiamen, PR China; 5 The Center for Evidence-based Medicine, Fudan University, Shanghai, PR China

**Keywords:** ultra-low vision, visual function questionnaire, rasch model, reliability

## Abstract

**Purpose:**

To develop a short form of Chinese ULV-VFQ-50 based on the ULV-VFQ-150 and compare the psychometrical properties of the two questionnaires.

**Methods:**

We selected candidate items from the ULV-VFQ-150, considering the item response among ultra-low vision (ULV) participants, the even distribution of item measures, visual aspects, and visual domains, to construct a 50-item ULV-VFQ-50 questionnaire. Then, ULV participants were recruited to evaluate its psychometric characteristics by using Rasch analysis.

**Results:**

In total, 79 out of 79 completed questionnaires of ULV-VFQ-50 were collected, of which 11 filled questionnaires were excluded because the participants' vision did not meet the inclusion criteria. Thus 68 valid questionnaires were analyzed (valid response rate 91.9%). The average age of the eligible responders was 45.0 years (standard deviation [SD] = 16.7), with 42.6% females (29/68). As per Rasch analysis, the person measures ranged from −1.74 to 4.91 logits, and the item measures ranged from −1.56 to 1.15 logits. The mean value of item difficulty was 0.00 logits, whereas the mean value of personnel distribution was −0.35 logits. The item reliability was 0.95, and the person reliability was 0.98. The items conform to unidimensionality as indicated by principal component analysis of the residuals, which showed that the first principal component unexplained only 5.5% of the total variance, and each component after that unexplained even less.

**Conclusions:**

The Chinese ULV-VFQ-50 exhibits excellent psychometric properties. The short form of Chinese ULV-VFQ, with fewer items and less administration time is better suited for clinical practice and research settings.

**Translational Relevance:**

The Chinese version of ULV-VFQ-50 is a reliable assessment of the visual function for people with ULV in China.

## Introduction

Ultra-low vision (ULV) describes a condition where individuals have severely impaired vision, allowing only the perception of light, movement, and basic shapes but not detailed forms. As individuals with ULV struggle to perform daily tasks that necessitate higher visual acuity, traditional visual functioning assessments are often inadequate for evaluating their visual capabilities.^1^ Specialized tools like the ULV Visual Functioning Questionnaire (ULV-VFQ) have been developed to address this problem. The ULV-VFQ is tailored to measure the visual abilities of those with profound vision loss, including tasks that can be performed with minimal vision. The ULV-VFQ was created to fill the gap left by conventional questionnaires, focusing on activities that individuals with ULV can relate to and perform. It ensures accurate measurement of visual abilities through psychometrically validated items, offering a reliable way to assess the functional impact of severe visual impairment. The original 150-item questionnaire has been streamlined into shorter versions with 50, 23, and 17 items, making it more feasible for clinical use while retaining its effectiveness and reliability (see Supplementary Material 1).^2^^–^^4^

We have developed a Chinese version of the Ultra-Low Vision Visual Function Questionnaire-150 (ULV-VFQ-150) and validated its psychometric properties.^5^ However, the lengthy time required to complete the 150-item questionnaire (approximately 40 minutes to one hour) limits its practicality, especially in time-sensitive research scenarios like clinical trials.^5^ Thus a clear need arises for a shorter version of the ULV-VFQ that maintains its comprehensive psychometric strengths without significant compromises. This endeavor aims to streamline the assessment process, enhancing the questionnaire's efficiency and making it more feasible for clinical trials and other research activities.

In this context, the development of the Chinese ULV-VFQ-50 is a strategic response to these challenges, particularly the need to reduce the administration time of the assessment. The full ULV-VFQ-150, while comprehensive, requires a significant amount of time to administer—often 40 minutes to an hour—which is burdensome for ULV individuals on one hand and impractical in many Chinese clinical and research settings, where time is a limiting factor on the other hand. By selecting and refining 50 items from the extensive ULV-VFQ-150, the goal was to create a more practical tool for evaluating the visual abilities of individuals with ULV and significantly reducing the administration time to approximately 20 minutes. This condensed version aims to retain the depth of patient experience insights while improving usability in fast-paced clinical and research environments. The ULV-VFQ-50's development process involved rigorous item selection and psychometric validation, including Rasch modeling, to ensure that it remains a reliable and valid tool for assessing visual function and its impact on quality of life. This effort represents a critical step toward enhancing the accessibility and applicability of visual function assessments, providing a valuable resource for clinicians and researchers dedicated to understanding and improving the lives of those with severe visual impairments.

## Material and Methods

### Data Source and Study Population

Between July 2022 and December 2022, the study aimed at recruiting potential participants with ULV from various sources, including Zhongshan Hospital affiliated to Fudan University, rehabilitation centers, and nursing homes located in Nantong, Jiangsu Province, as well as the Patient Assistance Group for Retinitis Pigmentosa in China. The ethical approval for this study was granted by the Ethics Committee of Zhongshan Hospital, Fudan University, under the approval letter numbered B2021-252R. This research strictly followed the ethical guidelines outlined in the Declaration of Helsinki, ensuring respect and protection for the participants involved. The participant eligibility criteria mirror those set forth in prior research, specifically: (1) individuals aged 18 and above; (2) native speakers of Chinese; (3) absence of cognitive impairments; and (4) a visual acuity range from counting fingers, hand motions, and light perception up to a maximum of 20/500 in the better-seeing eye, as verified by their medical documentation.^5^

In this study, the cognitive status of participants was informally assessed during each interview by evaluating the coherence, logic, and relevance of their verbal responses. Interviewers closely monitored the participants’ ability to understand questions, respond appropriately, and maintain logical consistency throughout the conversation. This conversational approach was used as a practical cognitive screening method, ensuring that participants had sufficient cognitive function to complete the questionnaire reliably.

The study measured visual acuity for patients enrolled during outpatient visits using the Snellen chart, a widely used tool in clinical settings. Participants were assessed using best-corrected visual acuity under consistent lighting conditions, and repeated measurements were taken to ensure accuracy. For patients from other regions who were unable to attend our hospital's outpatient visual acuity tests in person, we relied on their outpatient medical records from the past five years. Specifically, we included only those with at least three medical records, each meeting the inclusion criteria for the study. If the visual acuity results from different visits varied, we calculated the average value to ensure a more accurate representation of the patient's visual status.

### Development Procedures

In this study, we referred to the simplified process of the English version of the ULV-VFQ-150 study conducted by Dagnelie et al.^2^ When developing the Chinese version of the 50-item ULV function questionnaire, we adhered to the following principles:

1. In the process of developing the Chinese version of the 50-item ULV function questionnaire, we rigorously adhered to a structured methodology to ensure comprehensive coverage and relevance. The original 150-item questionnaire meticulously spans multiple independent visual aspects, including contrast (93/150), with the highest representation, followed by aspects such as lighting (18/150), motion perception (9/150), distance(8/150), and familiarity (5/150), environmental lighting (3/150), size (3/150), and other minor aspects (11/150).^5^ Recognizing the varied difficulty levels among these aspects, particularly noting the challenges posed by size, distance, and familiarity versus the relative ease of lighting-related items, the development prioritized a balanced representation to ensure that the difficulty levels of the selected items from the full instrument were matched and maintained in the 50-item version.^3^
2. Furthermore, ULV-VFQ-150 extends to encompass several visual domains, notably reading (3/150), information gathering (107/150), visual motor (30/150), and mobility (10/150), acknowledging prior findings that reading tasks are notably challenging for individuals with ULV.^5^ This inclusion ensures the questionnaire's capacity to evaluate a wide spectrum of visual function domains, and these visual domains remain consistent between the 50 and 150-item versions.
3. In the Chinese version of ULV-VFQ-150, the item measure (IM) span of the items was about 3 logits, and many items were similar in difficulty.^5^ The IMs span in the ULV-VFQ-50 was carefully considered to preserve the broad difficulty range while eliminating redundancy. This strategy allows for assessing a wide range of abilities, ensuring the questionnaire's applicability across diverse patient profiles.
4. Last, the selection of items for the simplified version was informed by analyzing response rates and cultural applicability from previous studies, prioritizing items that demonstrated higher engagement and relevance. This approach ensures that the simplified questionnaire remains sensitive to patient experiences and is culturally appropriate, enhancing its utility and effectiveness in assessing ULV functions.

### Rasch Model

For this study, the responses were scored on 1  =  impossible to see or do visually; 2 = very difficult; 3 = somewhat difficult; 4 = not difficult, with a specific category designated for “not applicable” responses, treated as missing data. The analytical framework employed the Andrich polytomous model for joint maximum-likelihood estimation.^6^ The choice to use it in this study is driven by its suitability for analyzing data with ordinal response categories, common in questionnaires like ULV-VFQ-50. This model is a prominent variant within the family of polytomous Rasch models and enables the detailed examination of responses across multiple categories. The main advantage of this model lies in its ability to maintain a consistent threshold structure across items while allowing for the relative distance between these thresholds to be the same, despite differing item difficulties. This nuanced approach facilitates a more accurate representation of both respondent abilities and item characteristics on a common metric scale, enhancing the interpretability and utility of the measurement outcomes.^6^^,^^7^

The Rasch model, foundational in this analysis, facilitates a probabilistic interpretation of data derived from surveys and tests, aiming to measure latent traits such as abilities or attitudes. It is distinguished by its capacity to concurrently calibrate item difficulty and person ability on a common scale. It enables a direct comparison and provides insights into the alignment between item challenges and respondent capabilities.^8^ By using WINSTEPS (version 5.1.7.0; www.winsteps.com), key analytical components of this study included the following:

#### Person-Item Mapping

The Person-Item Map, also known as the Wright map, is a graphical representation used in Rasch analysis to depict the distribution of both person measures (PMs) and item measures (IMs) on the same logit scale. By aligning respondent abilities with item difficulties, this visual representation provides an overview of how well the items span the range of participant abilities. In our study, the Wright map is used to analyze and present the alignment between the difficulty of the ULV-VFQ-50 items and the visual function levels of the participants.^9^

#### Anchoring IMs

Anchoring in Rasch analysis refers to fixing certain item measures based on prior knowledge or data, allowing for direct comparison across different datasets or test administrations. In this study, anchoring was applied to IMs during the analysis of the ULV-VFQ-50 in Round 1 to ensure consistency with the original ULV-VFQ-150 data. This method helps maintain the stability of item difficulty estimates across different versions of the questionnaire.^10^

#### Fit Statistics

Within the framework of the Rasch model, both ability and difficulty are regarded as latent variables (i.e., constructs that are not directly observable but can be estimated through fit statistics aligned with the model).^11^ These fit statistics refer to the infit mean square statistics, which indicate how well individual item responses align with the expectations set by the Rasch model. The Z-scores represent the standardized residuals between observed and expected responses. In Z-std bubble charts, Z-scores above 4 typically signal a lack of fit, indicating that the item responses deviate significantly from the model's expectations.^2^ Such instances may suggest either inappropriate responses from participants or potential issues with the content being measured by the questionnaire. These statistics evaluate how well the data fit the Rasch model expectations, with separate metrics for items and persons to identify outliers or misfit elements.^12^^,^^13^

#### Principal Component Analysis (PCA)—Unidimensionality

PCA investigates the dimensionality of the dataset, ensuring that the items measure a single underlying construct, which is crucial for the validity of the Rasch model.^14^ The principal component ought to account for the majority of variance. For the scale to be considered unidimensional, the raw data must explain at least 60% of the variance, and the first residual component's unexplained variance should not exceed 10%.^14^

#### Category Probability Curves

These curves depict the likelihood of a response falling into each category based on the trait level, highlighting the appropriateness of response categorizations and their thresholds.^6^^,^^15^

#### Differential Item Functioning (DIF) Analysis

DIF analysis assesses whether different demographic groups (e.g., age, gender) respond differently to items, indicating potential biases.^10^ The combined group was split in different ways in separate analyses to assess four possible bases for DIF: (1) online versus telephone administration, (2) gender (male vs. female), (3) retinitis pigmentosa versus non- Retinitis pigmentosa, and (4) age (>45 years vs. ≤45 years, because the mean age of the participants was 45 years old). We used the Bonferroni correction in this context to mitigate the risk of type I errors, which occur when a true null hypothesis is incorrectly rejected. Given that multiple statistical tests were conducted simultaneously across 50 items, the likelihood of encountering false positives increases proportionally with the number of tests. The Bonferroni correction is a conservative statistical adjustment used to maintain the overall error rate at a desired significance level by dividing the original alpha value (0.05) by the number of comparisons (50 in this study). This adjustment ensures that the findings are robust and reduces the probability of falsely identifying items as exhibiting DIF when they do not, thereby upholding the integrity of the psychometric analysis, so the *P* value for statistical significance was adjusted from 0.05 to 0.001.^10^^,^^16^^,^^17^

By using these rigorous methodologies, the study aimed to ensure the ULV-VFQ-50's reliability, validity, and comprehensive assessment capability, providing nuanced insights into the visual function of individuals with ULV.

### Instrument Development—Subset Construction

We performed the following steps:

1) Two researchers, C.J. (researcher A) and XY.W. (researcher B), independently reviewed the ULV-VFQ-150. Researcher A identified candidate items based on the item measure (IM) distribution depicted in the ULV-VFQ-150 Wright map and the patient response rates to each item from our previous study.^5^ This initial step resulted in the retention of 17 items, specifically item numbers 123, 115, 112, 59, 94, 113, 130, 74, 129, 70, 90, 1, 39, 96, 22, 145, and 84.
2) Researcher A focused on selecting items infrequently marked as “not applicable” and straightforward for participants to understand based on the response rates to the Chinese version of the ULV-VFQ-150. The selected items had a response rate of 90% (54/60) or higher, including items 2, 9, 10, 13, 16, 22, 30, 41, 42, 44, 48, 52, 64, 66, 68, 71, 88, 92, 97, 100, 107, 111, 116, 121, 131, 136, 140, and 147 (see Supplementary Material 2). Consequently, this phase resulted in the retention of the aforementioned 28 items.
3) Considering the classification into visual aspects and domains, it was noted that among the 45 items currently retained, there were only two items addressing “familiarity” and “size” within visual aspects, and a single item addressing “reading” within visual domains. Therefore it became essential to appropriately augment items related to “familiarity,” “size,” and “reading” to ensure a comprehensive evaluation. In light of a detailed evaluation of patient response rates and the relevance to cultural contexts, an additional five items were selected for inclusion: item 3 (familiarity, information gathering), 60 (familiarity, information gathering), item 93 (size, information gathering), item 119 (familiarity, information gathering), and item 124 (distance, reading). These additions were aimed at enhancing the representativeness of these critical visual aspects and domains within the questionnaire, ensuring a thorough assessment of visual function. Following the above meticulous three-step selection process, researcher A finalized the retention of 50 items, specifically 1, 2, 3, 9, 10, 13, 16, 22, 30, 39, 41, 42, 44, 48, 52, 59, 60, 64, 66, 68, 70, 71, 74, 84, 88, 90, 92, 93, 94, 96, 97, 100, 107, 111, 112, 113, 115, 116, 119, 121, 123, 124, 128, 129, 130, 131, 136, 140, 145, and 147. These items were chosen based on their coverage of crucial visual aspects and domains, including “familiarity,” “size,” and “reading,” while also considering patient response rates and cultural applicability. This curated selection forms the Chinese version of the ULV-VFQ-50-A.
4) Following the same meticulous procedure, researcher B also conducted an independent thorough review based on the response rate, item measures (IMs). The final selection included items 1, 2, 3, 9, 10, 13, 16, 22, 30, 39, 41, 42, 44, 48, 52, 59, 60, 64, 66, 68, 70, 71, 74, 84, 88, 90, 92, 93, 11, 96, 97, 100, 107, 111, 112, 127, 115, 116, 119, 121, 123, 124, 128, 129, 141, 131, 136, 140, 145, and 147. These carefully chosen 50 items form the second Chinese version of the ULV-VFQ-50-B.
5) Researchers A and B, on reviewing the two Chinese versions of ULV-VFQ-50, identified only three items that differed between the two versions. Specifically, ULV-VFQ-50-B included item 11, which inquires, “How difficult is it to see a bright icon on an iPad or tablet in a dark room?” This item was chosen based on the Wright map distribution results from step 1 and had a response rate of 53 out of 60. In contrast, ULV-VFQ-50-A opted to retain item 94, asking, “How difficult is it for you to see white rice on a white plate?” which had a slightly higher response rate of 55 out of 60. In addition, ULV-VFQ-50-B included item 127, which asks, “When visiting someone in a hospital, how difficult is it to see a pale-skinned, elderly person in a light-colored hospital gown lying in bed?” with a response rate of 53 out of 60. On the other hand, ULV-VFQ-50-A selected item 113 ( When walking through the woods on a sunny day, how difficult is it for you to see a stray dog from 10 meters away?), which had a response rate of 52 out of 60. This selection difference further illustrates the careful consideration of specific visual scenarios and the response rates to these items, reflecting the aim to encompass a wide range of visual experiences and challenges within the questionnaire. Last, ULV-VFQ-50-B chose to include item 141, which queries, “When standing at a black countertop, how difficult is it for you to notice a white pill lying right in front of you?” with a response rate of 53 out of 60. Conversely, ULV-VFQ-50-A opted for item 130, asking, “When standing at a white countertop, how difficult is it for you to notice a dark pill lying right in front of you?” which received a response rate of 52 out of 60.
6) After a comprehensive team discussion, it was decided that all 53 items identified would be included in the questionnaire survey for further evaluation. This decision was made to ensure a thorough assessment of each item's relevance and effectiveness in capturing the visual functioning of individuals with ULV. Upon completing the questionnaire survey, the items with higher response rates were selected to finalize the Chinese version of ULV-VFQ-50. The interviews revealed that items 94, 113, and 130 had response rates of 61/68, 60/68, and 63/68, respectively, whereas items 11, 127, and 141 garnered higher response rates of 63/68, 65/68, and 67/68, respectively. Based on these findings, items 11, 127, and 141 were chosen for retention due to their superior response rates.

The items finally retained to form the final Chinese version of ULV-VFQ-50 are 1, 2, 3, 9, 10, 11, 13, 16, 22, 30, 39, 41, 42, 44, 48, 52, 59, 60, 64, 66, 68, 70, 71, 74, 84, 88, 90, 92, 93, 96, 97, 100, 107, 111, 112, 115, 116, 119, 121, 123, 124, 127, 128, 129, 131, 136, 140, 141, 145, and 147. This refined list ensures the questionnaire comprehensively addresses various aspects of visual functioning, grounded in the item's relevance and participant engagement. To provide a clearer understanding of the questionnaire development process, a detailed flowchart is provided in Supplementary Material 3.

## Results

### Administration Time

In this study, the mean time to complete the Chinese ULV-VFQ-50 was 20.4 minutes (SD = 16.1), and the median time was 21.0 minutes (range 16.0–31.0 minutes). In contrast, the full ULV-VFQ-150 requires 40 minutes to one hour to complete.^5^ This indicates a notable reduction in the time participants needed to complete the questionnaire, reflecting the improved efficiency of the shorter version. The moderate variability in completion time for the 50-item questionnaire also reflects the diverse experiences and capabilities of the individuals involved.

### Participant Demographics

In the previous ULV-VFQ-150 study, we recruited a total of 60 patients with ULV, with the demographic data shown as “Round 1.”^5^ In the current 50-item study, out of 79 collected questionnaires, 11 were excluded because the participants' vision levels did not meet the study's inclusion criteria, leaving 68 valid questionnaires (valid response rate 86.1%), with the demographic data presented as “Round 2.” The median age of the 68 participants was 40.5 years, spanning from 19 to 87 years, with an average age of 45.0 years and a standard deviation of 16.7. The gender distribution included 29 female participants, making up 42.6%, and 39 male participants, accounting for 57.4%. Among these, 58.9% (40 out of 68) were diagnosed with retinitis pigmentosa.^5^

### Round 1—Survey Analysis

In the first round of the study, we used the dataset from the ULV-VFQ-150 study with 60 participants to examine the impact of reducing the number of items on the psychometric properties of the ULV-VFQ.^5^

#### Comparison of Wright Maps With Anchored IMs

**Figure 1.**
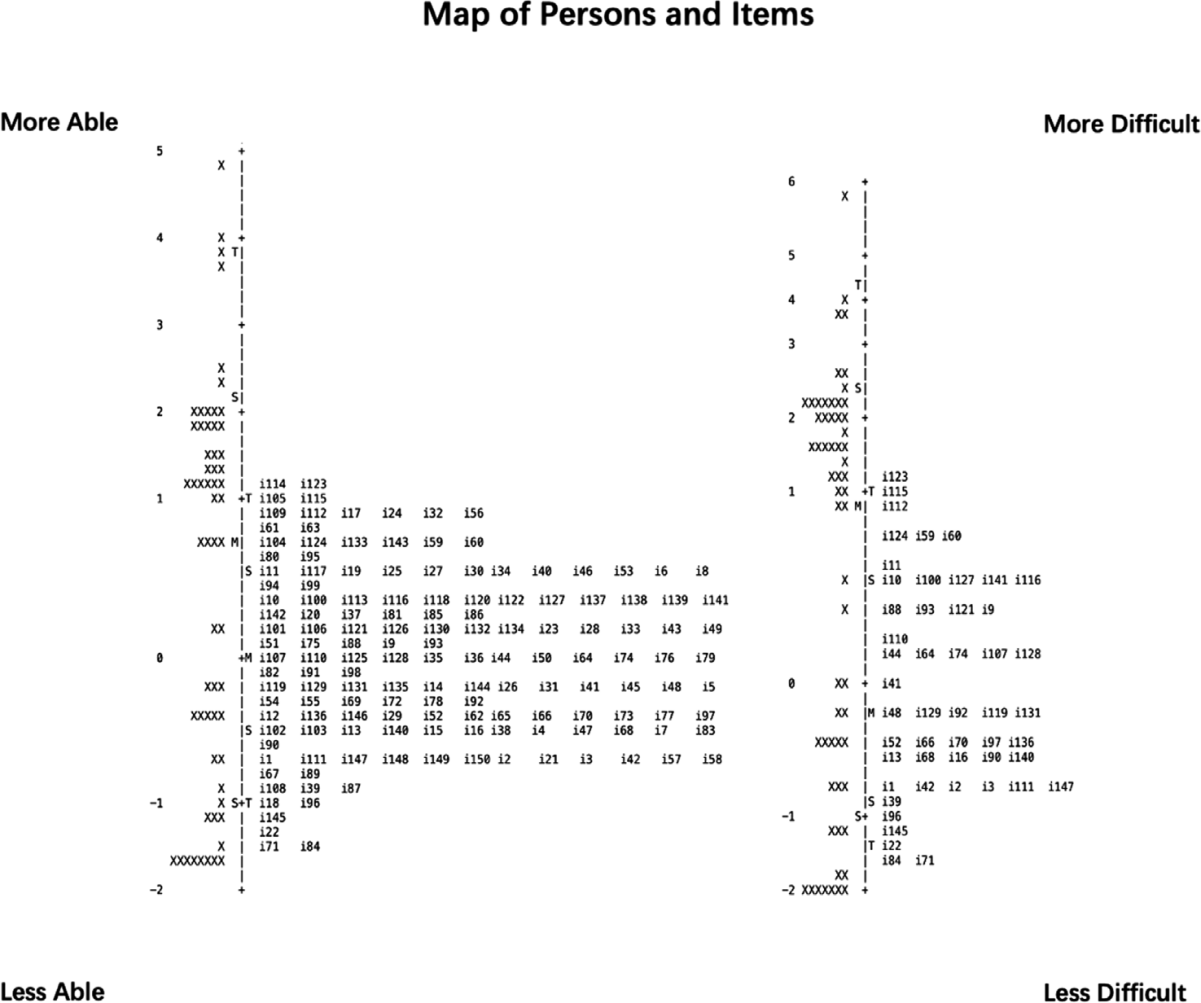
Map of Persons and Items of Round 1. Two Wright maps display Person-Item plots on shared logit scales for the ULV-VFQ-150 (*left*) and ULV-VFQ-50 (*right*), using item anchoring in analyses of the smaller item sets. Both item difficulty and person ability increase from the bottom upward.

As described earlier, the 50-item version was optimized for even distribution across the IM scale and for capturing various visual aspects and domains. Figure 1 illustrates the distributions of the person measures (PMs), which are denoted by ‘X’ to the left of the logit axis in each panel, and IMs, which are indicated by item numbers to the right of the logit axes. The logit axis in each case is marked with the mean IM (set to 0 by default) and PM, as well as markers for one standard deviation (S) and two standard deviations (T).

In both the Chinese 150-item and 50-item versions, the item measures are identical, adhering to the item anchoring in Rasch analysis, which involves fixing certain items with known difficulty levels to calibrate and compare results. This approach ensured that the item difficulty estimates remained stable across different versions. The distinction lies in excluding unused items before the analysis, with the 50-item set recording an IMs range from −1.56 to 1.15 logits, essentially maintaining the range of the 150-item version. Many IMs in the 150-item set are statistically indistinguishable from those of adjacent items, indicating redundancy, which is mostly resolved when trimmed to 50 items.

#### Correlation, Visual Aspects, and Functional Visual Domains

Table 2 delineates a comparative analysis between the 150-item and 50-item versions of the ULV-VFQ with the IMs anchored. It methodically presents metrics such as Item Measures (IMs) and Person Measures (PMs) in logits, showing subtle shifts in median values and ranges when the item set is condensed. Specifically, in the 50-item version, the median of the IMs decreases slightly, reflecting a more selective approach to item retention and resulting in tighter spacing between items. Meanwhile, the median of the PMs shows a slight increase in the 50-item version, possibly indicating that the retained items are generally more challenging. Although the reduction in the number of items leads to an increase in the standard errors (SE) of the PM estimates, this increase remains within expected limits, following the inverse proportionality to the square root of the item set size. This suggests that while reducing the number of items does introduce more uncertainty, it is controlled and manageable.

Notably, as the item count increases, a marginal dip in overall the reliability of the PMs is observed—with the 50-item version registering PM reliability of 0.98, contrasted with 0.99 in the 150-item format. Conversely, IMs reliability in the 50-item version enhances to 0.89 from 0.87, indicative of heightened scale reliability and estimation accuracy among most participants.

The table also methodically charts the item distribution across various visual aspects and domains, revealing a strategic curtailment in the 50-item variant. This careful reduction retains a balanced representation of crucial visual areas, ensuring the condensed version adeptly encapsulates the core dimensions of visual function assessment. Ultimately, this table eloquently encapsulates the nuanced adjustments undertaken in the transition from the 150-item to the 50-item ULV-VFQ, manifesting a meticulous optimization of item selection and distribution that upholds the tool's comprehensive assessment capabilities and integrity.

#### Item and Person Measure Fit Statistics

**Figure 2.**
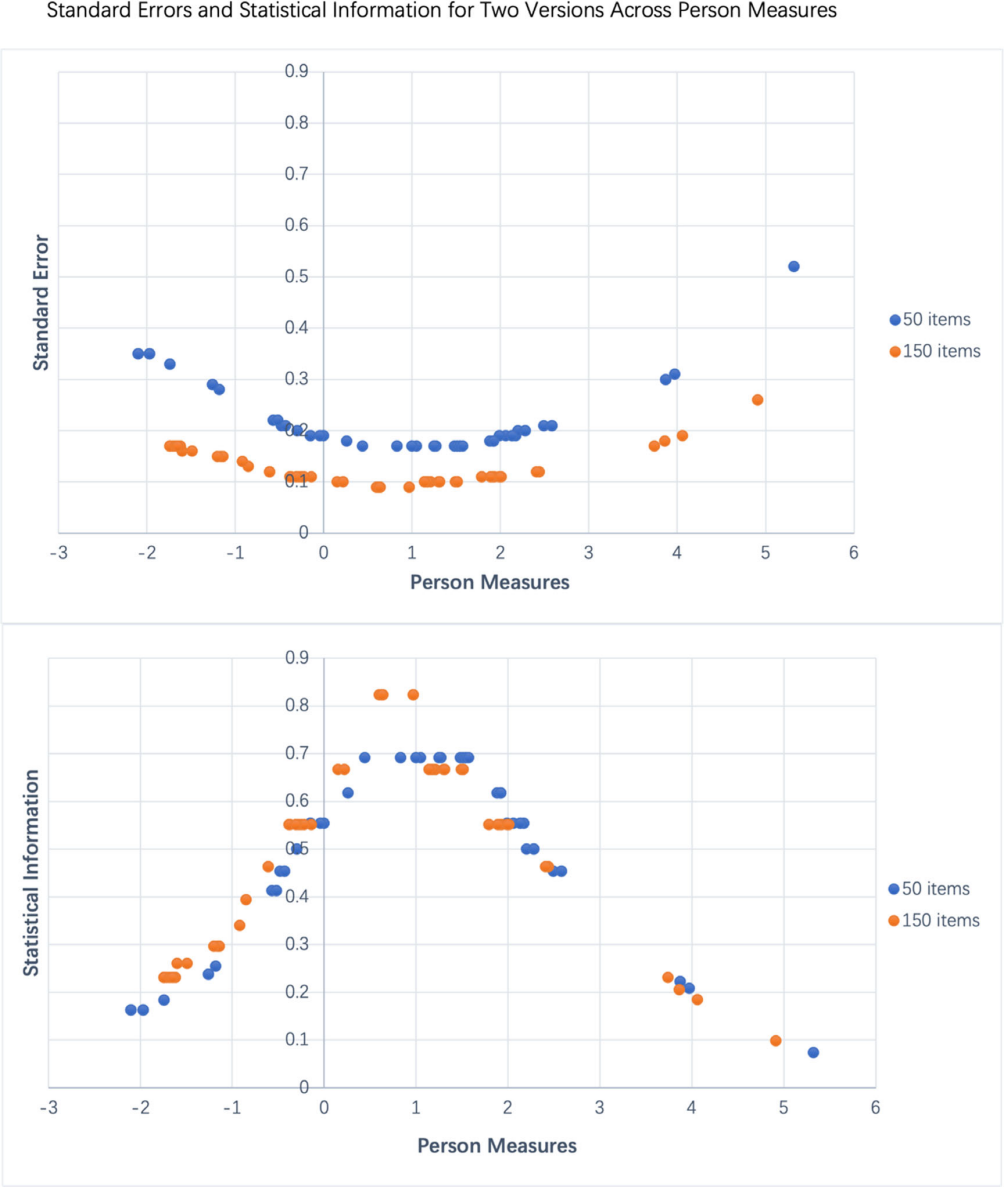
Standard error and statistical information for two versions across person measures: standard error (SE); *top*, statistical information ([N × SE^2^]^−1^; *bottom*) gained, as a function of PM, for the administration of two versions of the ULV-VFQ.

As Figure 2 shows, the relationship between SE and statistical information [N × SE^2^]^−1^, plotted against Person measures (PM) for item sets of 150 and 50, reveals nuanced dynamics in psychometric precision and the efficiency of information extraction. From the top chart, we can discern that as the number of items increases from 50 to 150, there's an increase in measurement precision—this is evident by the smaller SE for the 150-item set, especially prominent in the densely populated range of −1 to 2 logits. The SE is smallest at the center of the PM range, suggesting that the items are well-targeted for the average person's ability being measured. The application of fourth-order polynomial curves to the data illustrates a consistency in precision across the two sets, with a noticeable shift toward the center for smaller item sets. This highlights the complex balance in test design—where both the number and distribution of items are crucial to optimizing the assessment's precision and informational yield.

The bottom graph visualizes the statistical information obtained from the 50-item and 150-item sets, plotted against PM. It shows that, while the 150-item set provides a higher peak of information—indicating greater precision around the average ability level—the 50-item set maintains a relatively uniform level of information across all ability levels. This uniformity suggests that the items in the 50-item set are well selected to provide consistent insight into the measured traits across a wide range of PM values, illustrating an effective balance between quantity and quality in item selection for psychometric testing.

**Figure 3.**
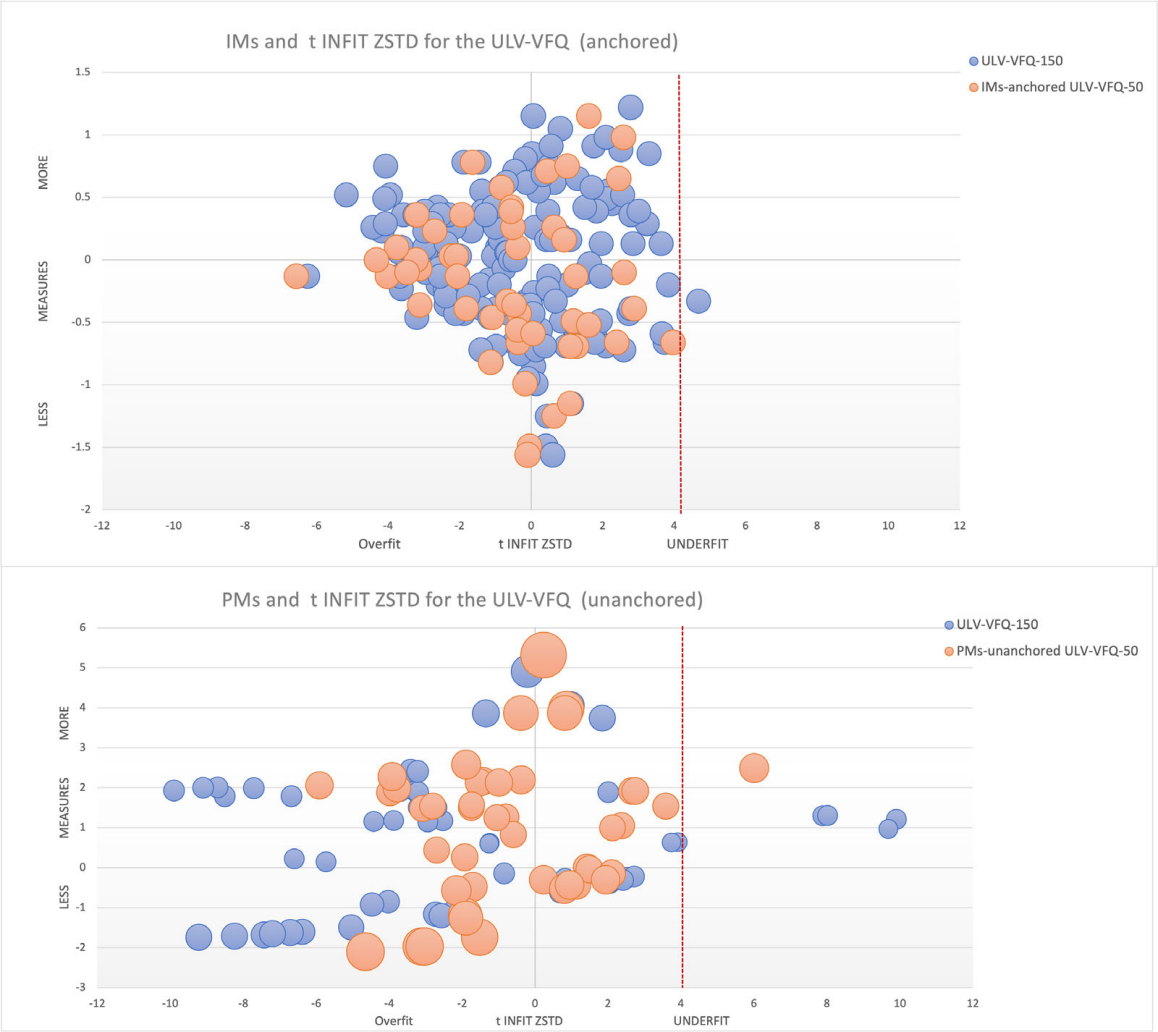
Bubble chart of Item and person ZSTD. IMs and reliability Z-scores (ZSTD) for the ULV-VFQ IMs (anchored; *top*) and PMs (unanchored; *bottom*). The bubble size in both plots corresponds to the standard error (SE) of the measure estimate.

The top part of Figure 3 illustrates anchored item bubble plots for the two versions of ULV-VFQ, distinguished by color coding to indicate each version. The single item that had a poor fit in the larger 150-item version of the ULV-VFQ is no longer included in the 50-item version.^5^ The bottom of the Figure 3 mirrors this, displaying overlaid PM bubble plots for both versions. The non-overlap of the bubbles reflects the PMs not anchored, in contrast to the items. It also shows that smaller sets tend to have fewer misfitting PMs. This trend is predictable as Z-scores inversely vary with the SE of the estimate, which is larger for the version with fewer items.

#### PCA

**Figure 4.**
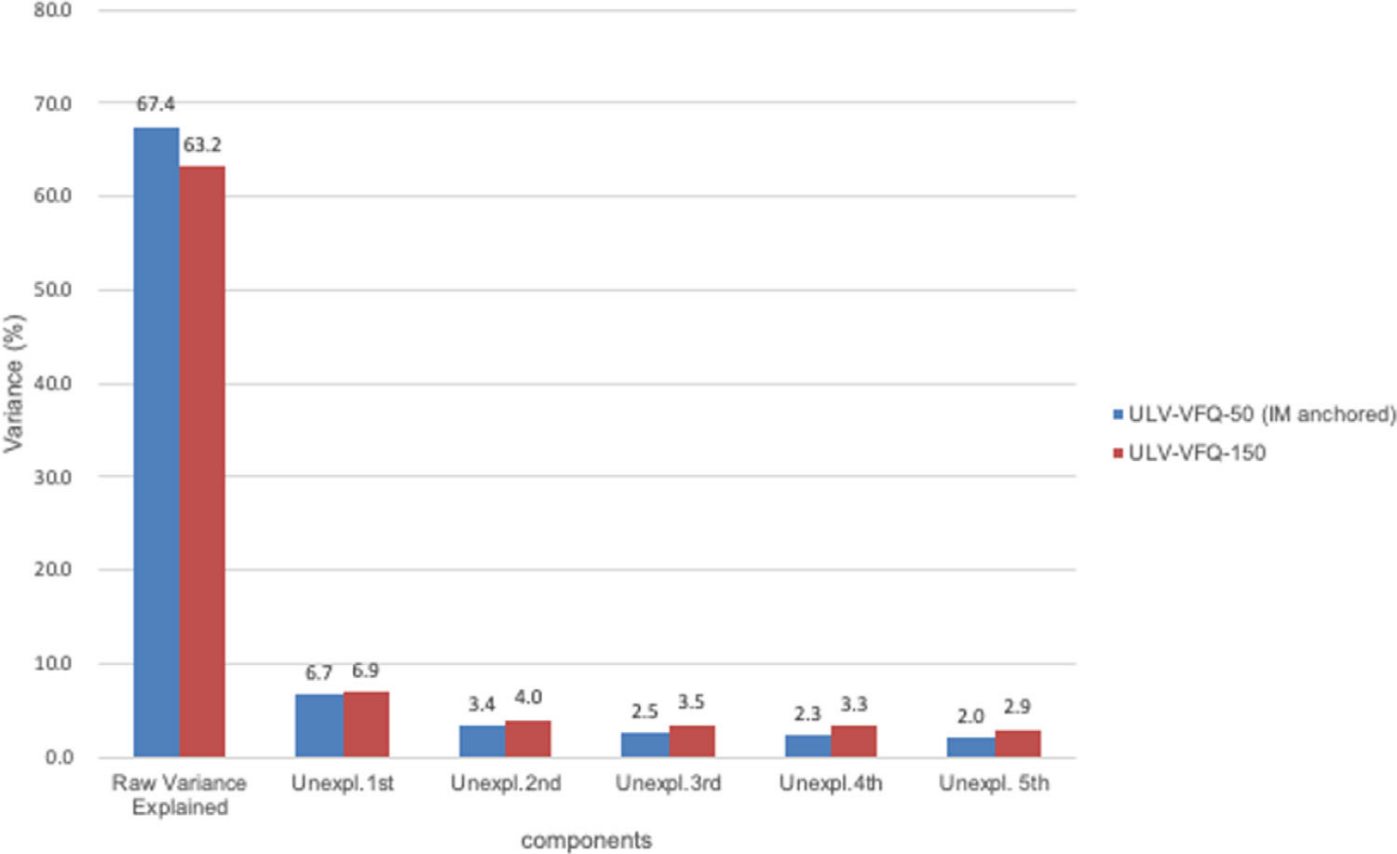
Principal component analysis of round 1.

PCA on residuals serves as a technique to assess a scale's unidimensionality.^14^ Figure 4 for ULV-VFQ-150 and ULV-VFQ-50 confirms this. The explained variances were estimated to be 63.2% and 67.4% for the 150 and 50 sets, and the first principal residual component contributes less than 10% to the total variance, aligning with unidimensionality criteria. Minimal residual variances further validate the scales' effective measurement of unidimensionality, consistent with Rasch model expectations.

### Round 2—Survey Analysis

In Round 1, we confirmed that the ULV-VFQ-50 exhibited good psychometric properties when anchored to the ULV-VFQ-150 item measures based on our previous 60-participant dataset.^5^ In round 2, we aimed to further validate its psychometric properties among a newly recruited group of 68 participants (Table 1^2^) with ULV who received interviews using the shortened questionnaire.

**Table 1.** Characteristics of Sample as n (%)

Demographic Data	Round 1 (n = 60)	Round 2 (n = 68)
Age (year)		
>45.0	25 (41.7%)	30 (44.1%)
≤45.0	35 (58.3%)	38 (55.9%)
Gender		
Male	39 (65.0%)	39 (57.4%)
Female	21 (35.0%)	29 (42.6%)
Group		
Telephone interviews	32 (53.3%)	41 (60.3%)
Online interviews	28 (46.7)	27 (39.7)
Visual Acuity (The better eye)		
VA 20/500	12 (20.0%)	10 (14.7%)
VA 20/667	10 (16.7%)	6 (8.8%)
VA 20/1000	1 (1.75)	3 (4.4%)
LP ≤ VA ≤ 20/2000	37 (61.7%)	49 (72.1%)
Diagnosis		
Retinitis pigmentosa	42 (70.0%)	40 (58.9%)
Other	18 (30.0%)	28 (41.1%)
Education		
Graduate student	1 (1.7%)	0 (0.0%)
Undergraduate	13 (21.7%)	15 (22.1%)
Junior college	10 (16.7%)	12 (17.6%)
High school	8 (13.3%)	9 (13.2%)
Junior high school	18 (30.0%)	19 (27.9%)
Primary school	8 (13.3%)	4 (5.9%)
Illiterate	2 (3.3%)	9 (13.2%)
Living situation		
Alone	7 (11.7%)	11 (16.1%)
Stayed with family	47 (78.3%)	48 (70.6%)
Did not respond	6 (10.0%)	9 (13.2%)
Number of going out to socialize a week		
> 3 times	8 (13.3%)	9 (13.2%)
2 to 3 times	34 (56.7%)	36 (52.9%)
≤ 1 time	12 (20.0%)	14 (20.6%)
Did not respond	6 (10.0%)	3 (4.4%)
Whether have obtained a driving license		
Yes	6 (10.0%)	5 (7.4%)
No	37 (61.7%)	41 (60.3%)
Did not respond	17 (28.3%)	22 (32.4%)

LP, light perception; VA, visual acuity.

**Table 2.** Comparison of the 2 Versions of the ULV-VFQ Discussed

Quantity and Measure	ULV-VFQ-150 (n = 60)	ULV-VFQ-50 (n = 60)
Item measure (logits)		
Median	0.03	−0.13
Min	−1.56	−1.56
Max	1.22	1.15
IM spacing (logits)		
Median	0.00	0.03
Min	0.00	0.00
Max	0.24	0.24
Person Measure (logits)		
Median	0.81	1.15
Min	−1.74	−2.10
Max	4.91	5.32
Person Measure SE (logits)		
Median	0.11	0.20
Min	0.09	0.17
Max	0.26	0.52
Mean*$\sqrt{N}$	1.76	1.61
Reliability		
IM	0.87	0.89
PM	0.99	0.98
Visual aspects		
Contrast	93	27
Luminance	17	7
Illumination	3	2
Familiar	4	4
Movement	8	2
Distance	11	2
Size	3	2
Other	11	4
Visual domains		
Reading	3	2
Mobility	10	4
Visual info	107	35
Visual motor	30	9

#### Person-Item Mapping

**Figure 5.**
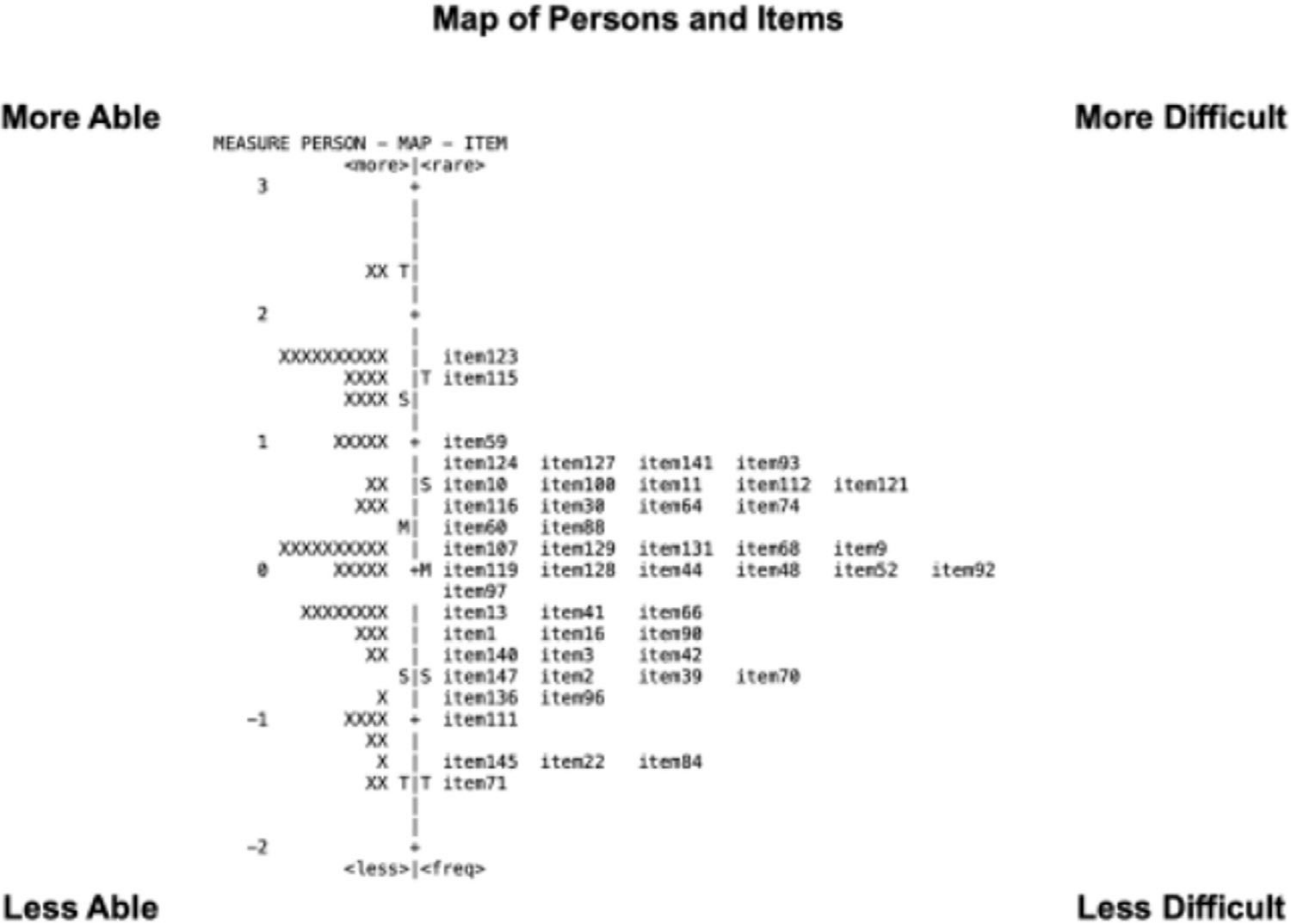
Map of persons and items of round 2.

In Figure 5, we observe that the person measures span a range from −1.6 to 2.4 logits, whereas the item measures extend from −1.6 to 1.7 logits. This range parity indicates that the 50-item version preserves the comprehensive assessment range found in the 150-item version,^5^ ensuring a similar spectrum of difficulty is evaluated. With a mean person ability offset of 0.37 logits from the conventional mean item difficulty at 0.0 logits, the items appear well targeted to the participants' ability levels. The mean difference between person abilities and item difficulties being close to or less than 1.0 logit suggests that the questionnaire effectively targets without significant mistargeting.^9^^,^^18^ The distribution of IMs across the PM range depicted in Figure 5 supports the notion that the questionnaire items align well with participants' visual abilities, such that the ULV-VFQ-50 is a valid alternative for the ULV-VFQ-150.

#### Category Probability Curve

**Figure 6.**
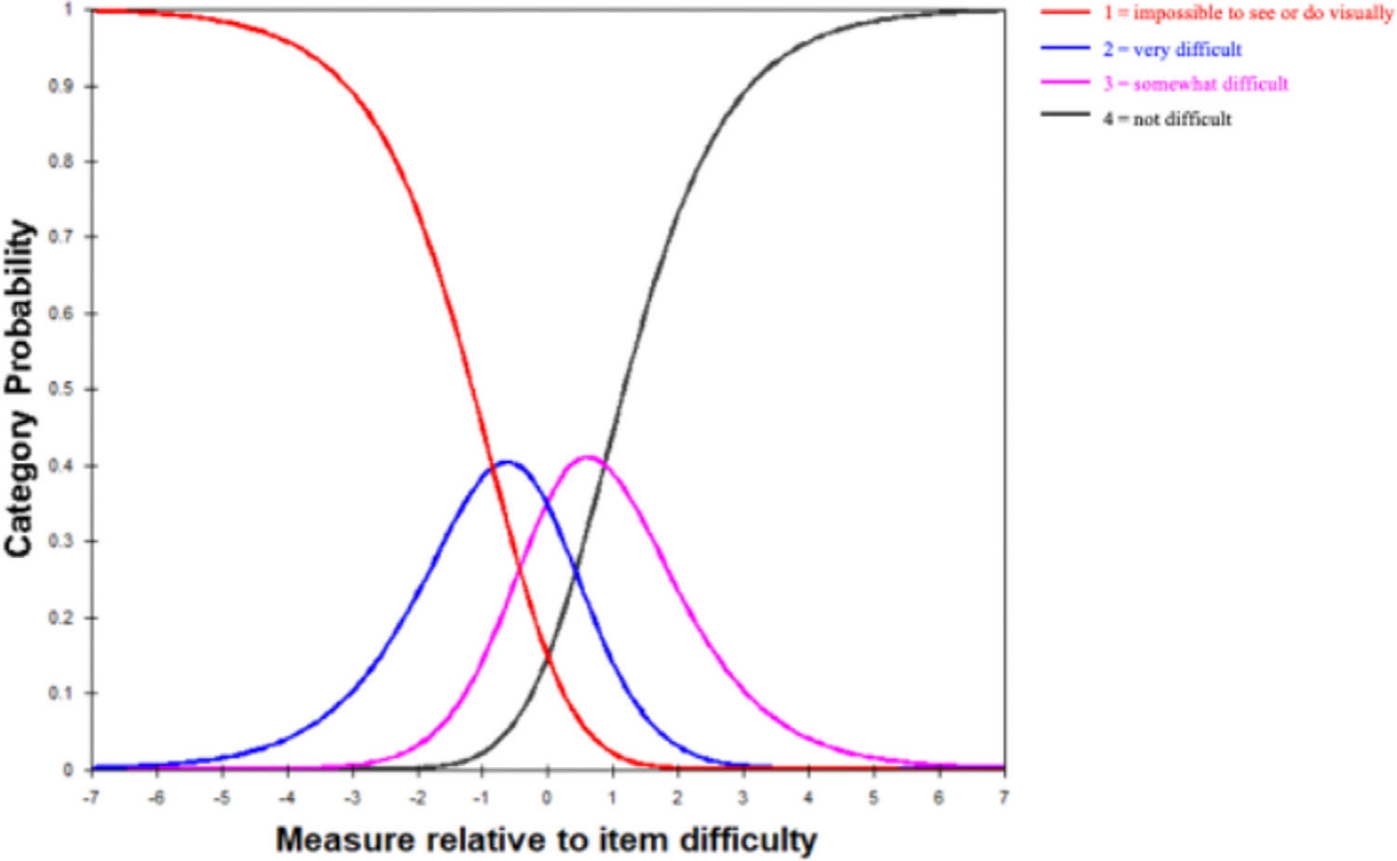
Category probability curves. The category probability curves illustrate the ordered threshold. The four curves from left to right represent four response categories (*red*: 1 = impossible to see or do visually; *blue*: 2 = very difficult; *purple*: 3 = somewhat difficult; *black*: 4 = not difficult).

The functionality of a rating scale can be visually appraised through a category probability curve graph, which delineates the probability of respondents selecting each response category across a continuum of an underlying trait.^19^ In Figure 6, the category probability curve graph indicates a well-structured rating scale, as evidenced by the absence of disordering in the response categories; each category has been uniformly selected by the respondents across the spectrum of measurement. The diagnostic output for the rating scale, as detailed in Table 3, supports this interpretation with all category frequency counts being substantial, thus facilitating the production of stable local estimates for the scale's structure.

**Table 3.** Analysis of the Rating Scale of Chinese ULV-VFQ-50

Category Label	Observed Count	Category %	Average Measure	Expected Measure	Infit Mean-Square	Outfit Mean-Square	Threshold Calibration
1	1568	47	−2.02	−2.02	1.05	1.06	None
2	873	26	−0.92	−0.89	0.90	0.75	−0.85
3	573	17	0.06	−0.02	0.96	1.13	−0.02
4	339	10	0.60	0.68	1.06	1.01	0.87

1 = impossible to see or do visually; 2 = very difficult; 3 = somewhat difficult; 4 = not difficult.

#### Fit Statistics

**Figure 7.**
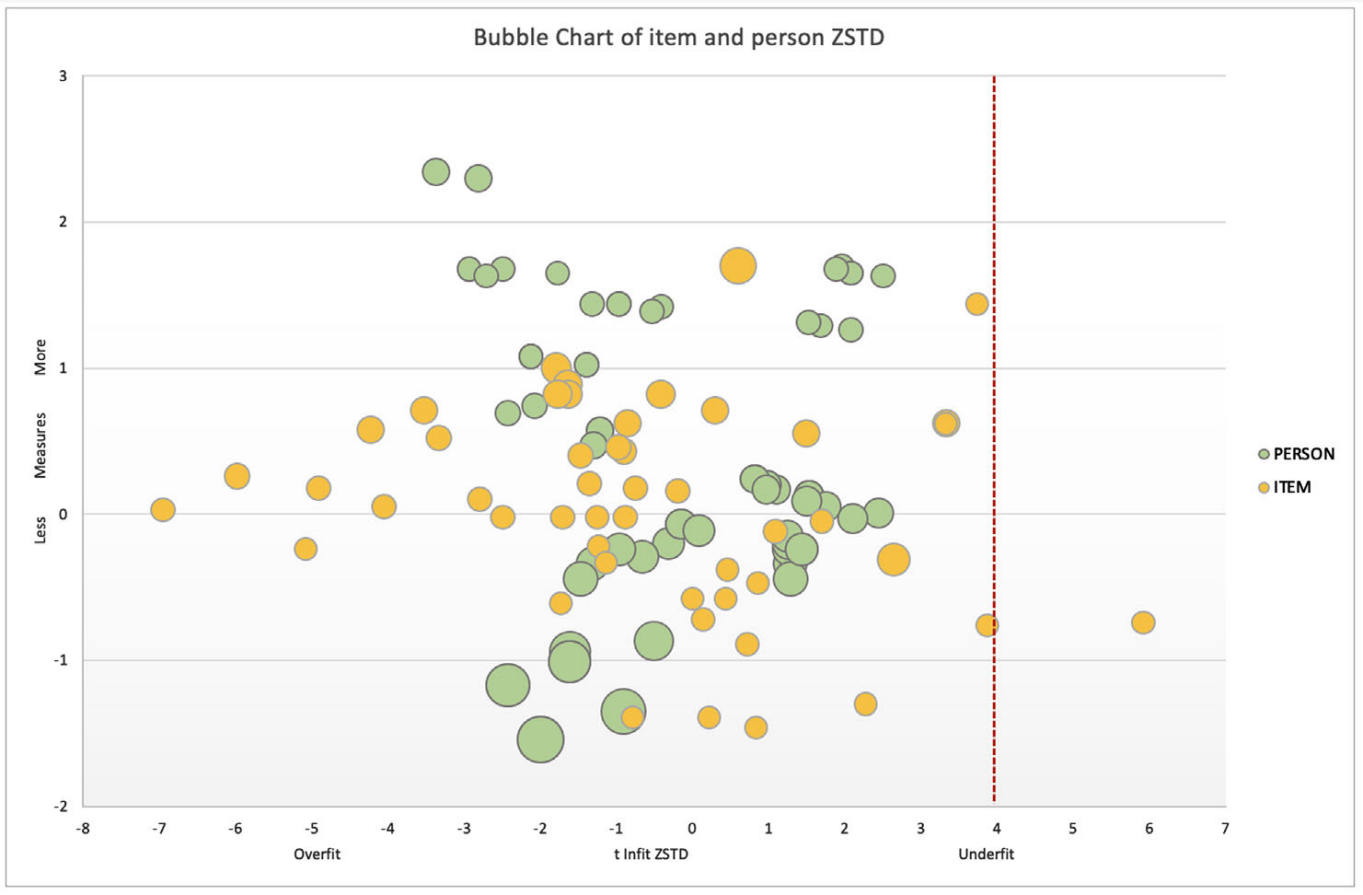
Bubble chart of item and person ZSTD.

Figure 7 effectively illustrates this concept. Notably, within the ULV-VFQ-50, one item that assessed the respondents' ability to identify the difference between khakis and dark denim jeans under fluorescent lighting—recorded a Z-std score above 4, indicating underfit. A significant proportion of the participants (58.9% or 40 out of 68) were diagnosed with retinitis pigmentosa, a condition known to degrade color contrast sensitivity, which likely contributed to the item's inadequate performance. Although items exhibiting underfit in the 150-item version were not retained for the 50-item version, the current round of analysis still identified an item with a Z-value exceeding 4.^5^ The reason for this might be linked to the specific challenges faced by participants with visual impairments in distinguishing subtle color contrasts. This particular item showed slightly poorer fit statistics compared to other retained contrast items (Supplementary Materials 4). Nevertheless, the overall fit statistics of the retained items in the questionnaire were favorable, demonstrating the instrument's robustness across different sets of contrast-related items.

Additionally, no participants in this study recorded a Z-std score greater than 4, reinforcing the model's capability to accurately predict visual functional responses within this group. This finding represents a significant improvement in fit over the previous study on ULV-VQF-150,^5^ validating the shortened questionnaire's effectiveness in visual functionality assessment.

#### Visual Domains and Aspects

**Figure 8.**
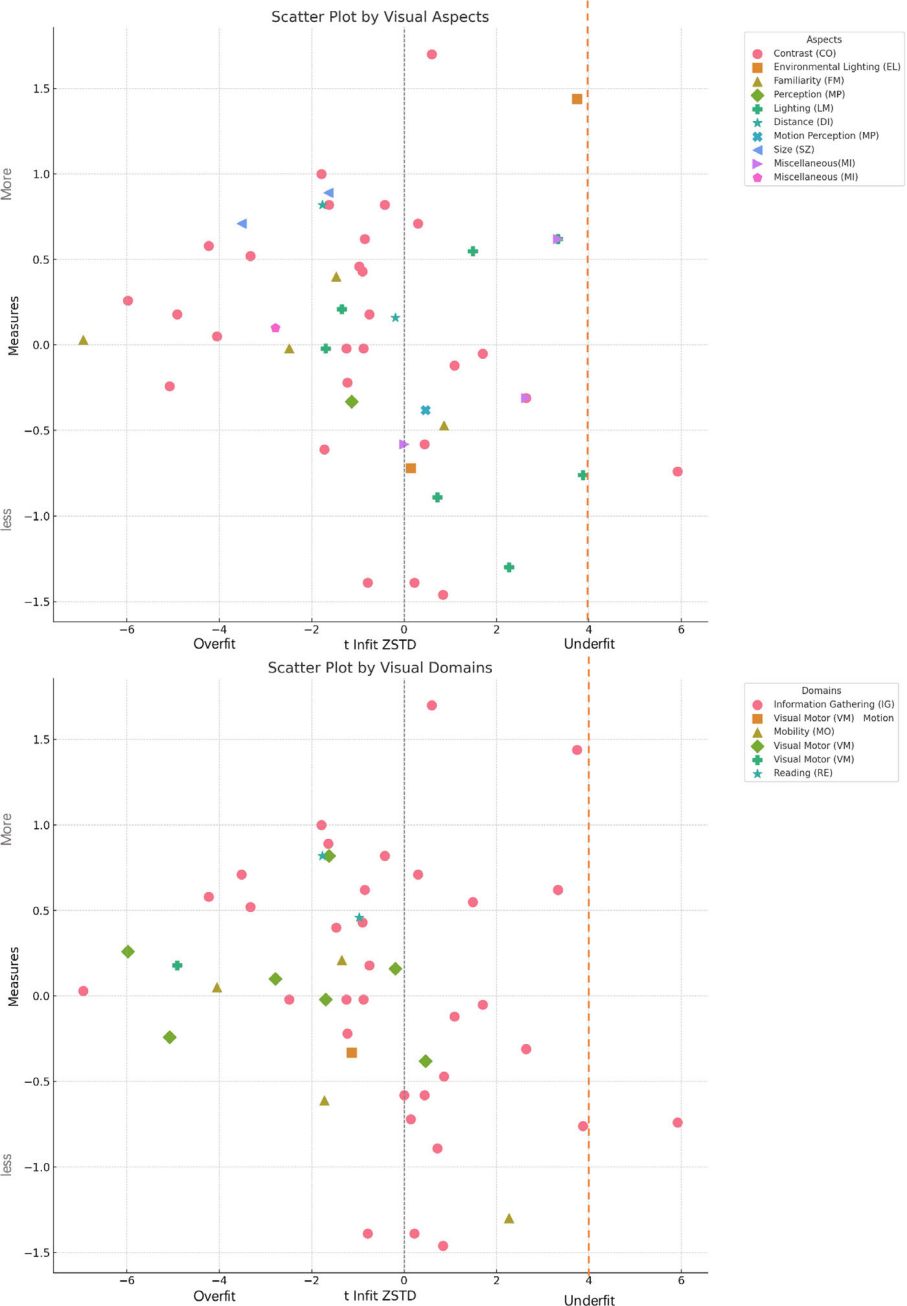
Item measures as visual aspects (N = 50; top) and domains (N = 50; bottom).

According to visual aspects and visual domains, item measures are color-coded in Figure 8. For visual domains, items associated with lighting were easier to determine (as indicated by lower item measures); in contrast, items determined by size and environmental lighting required more visual effort (as shown by higher item measures). Similar to the previous results, items with a broad range of IM values were influenced by contrast. It is also similar to the previous results that for the visual domains, items determined by mobility were easier (lower in the figure), whereas items determined by reading require more vision (higher item measures); items determined by information gathering show a wider range.^5^

#### DIF Analysis

In our DIF analyses, we assessed potential differences across the four predefined groups: online vs. telephone administration, gender (male vs. female), diagnosis (retinitis pigmentosa vs. non-retinitis pigmentosa), and age (>45 years vs. ≤45 years). After applying the Bonferroni correction for multiple comparisons, none of the items in the 50-item questionnaire showed statistically significant evidence of DIF (adjusted *P* value threshold = 0.001). This indicates that the items performed consistently across the different subgroups, suggesting that the shortened questionnaire is free from bias related to these factors and is a robust tool for measuring the intended latent traits across diverse populations.

#### PCA

**Figure 9.**
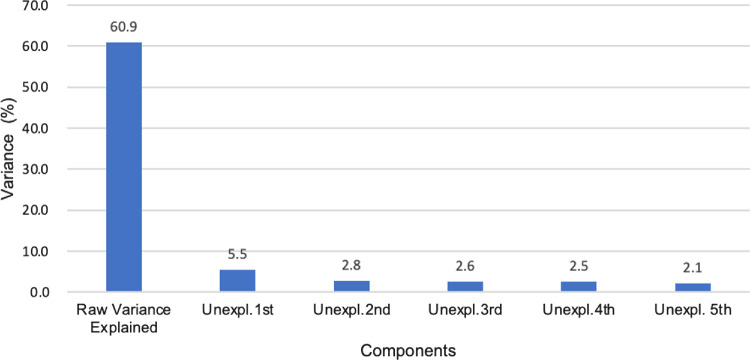
Principal component analysis of round 2.

Figure 9 shows that the 50-item scale has 60.9% explained variance, with the first residual principal component explaining only 5.5% of the total variance, which means that there was no major systematic deviation from the Rasch model and also adhered to unidimensionality.

## Discussion

Based on the Chinese version of ULV-VFQ-150,^5^ the current study aimed to develop a 50-item scale, the Chinese ULV-VFQ-50, which takes less time and is more suitable for clinical trials. This study kept all visual aspects and domains, as well as the range of IMs, from the original 150-item questionnaire as much as possible. Therefore, in developing the simplified version, the evenly spaced IMs entries were first retained, and all visual aspects and visual domains were also preserved.

In the psychometric evaluation of the shorter scale, the Wright maps show that, compared to the Chinese ULV-VFQ-150, the ability of persons is better matched with the difficulty of the items, mainly because the number of redundant items in the medium difficulty part is greatly reduced. In the Chinese ULV-VFQ-150, there is only one unfit item (Item 12: When entering a building, how difficult is to adjust from a bright sunny day to the dim interior lighting after five minutes?). This item was not retained in the 50-item scale. In the ULV-VFQ-50 fitting analysis, there was an unfit item to test persons' contrast sensitivity. Nearly 60% (40/68) of participants in the second round of interviews were patients with retinitis pigmentosa, who generally had poorer contrast sensitivity. We hypothesized that this poorer contrast sensitivity might contribute to the misfit of certain items. However, it is important to note that DIF based on contrast sensitivity was not explicitly analyzed in this study due to data limitations. Additionally, our overall DIF analysis found no significant DIF related to Retinitis pigmentosa status, indicating that despite the observed item misfit, the majority of items measured consistently across different groups.

As seen from the Wright map, most items are moderately difficult and are located in the middle of the scale. However, to investigate the visual ability of people with ULV to the greatest extent, it is also necessary to preserve the items at the extremes of IMs, because in the range of ULV, the ability of persons of relatively good visual ability and relatively poor visual ability is captured by marginal items.

Beside assessing the shorter version of the instrument by using the dataset of the Chinese ULV-VFQ-150 study,^5^ we also recruited 68 patients with ULV for its psychometrical evaluation, including 18 subjects who had responded to the 150-item questionnaire in the previous study. The 18 participants’ responses to the shared items in both questionnaires were not exactly the same. We detected some potential trends of the difference. Participants with retinitis pigmentosa showed more variability in responses, particularly for items related to contrast sensitivity, where 65% of patients with retinitis pigmentosa fluctuated between difficulty levels across different rounds of interviews. Additionally, younger participants and those with less than five years of visual loss exhibited more inconsistent responses compared to older participants and those with a longer duration of impairment. These differences suggest that factors such as disease progression and adaptation to vision loss contribute to the variability.^20^ In addition, the Chinese version of ULV-VFQ-50 was proved to be a single-dimensional scale, indicating that there are no meaningful subscales to distinguish in this scale.

The current research has some limitations. Among the 68 respondents of round 2, 58.9% (40/68) had retinitis pigmentosa. Therefore it needs to be clarified to what extent the findings of this study can be generalized to other patients with ULV.

The Chinese and English versions of the ULV-VFQ-50 both provide reliable and efficient assessments of visual function for individuals with ULV. Both versions were condensed from the original 150-item questionnaires to maintain a wide range of difficulty and comprehensive coverage of visual aspects and domains, validated through Rasch analysis. In comparing the Chinese and English versions of the ULV-VFQ-50, it is evident that there are both commonalities and unique elements in each version. Out of the 50 items, 12 items are identical in both versions, representing a 24% overlap. These common items include the difficulty of recognizing a freshly painted white crosswalk on dark pavement on a cloudy day and the challenge of seeing if a cell phone screen is on in a dark room. These shared items ensure consistency in assessing visual functions across different cultural contexts. However, there are significant differences as well. For example, the English version includes items specific to cultural and environmental contexts, such as “When watching an ice hockey game on a large TV, how difficult is it to see in which direction the players wearing dark uniforms are moving?” The items reflect activities more common in Western settings. In contrast, the Chinese version includes culturally relevant items like “When walking through the park on a sunny day, how difficult is it to see a stray dog from 10 meters away?” instead of a deer, as mentioned in the English version. This adaptation ensures the questions are more relatable to Chinese participants.

A comparison of psychometric measures showed that the item reliability in the Chinese ULV-VFQ-50 is 0.89, slightly lower than the 0.98 in the English 50-item version. Although this indicates a minor difference in consistency, both versions remain highly reliable, demonstrating robust effectiveness in assessing visual function in individuals with ULV. The person reliability is 0.98 for both the Chinese and English 50-item versions, indicating consistently high reliability across both sets.

Additionally, both versions show strong unidimensionality because the first residual component in the principal component analysis are both below the 10% threshold. The explained variances also confirm the scale's unidimensionality and effective measurement properties.

In summary, the Chinese ULV-VFQ-50 exhibited excellent psychometric properties and had an average completion time of 20.4 minutes, whereas the ULV-VFQ-150 typically requires between 40 minutes to one hour to complete. This marked reduction in administration time makes the ULV-VFQ-50 a more practical tool for both clinical and research settings, where time efficiency is crucial. The shorter completion time not only eases the burden on patients with ULV, who may find lengthy assessments fatiguing, but also enhances the feasibility of incorporating visual function assessments into routine care and large-scale studies. Therefore the improved time efficiency of the ULV-VFQ-50 supports its broader adoption and use as a reliable and valid measure of visual function in ULV populations. The consistent psychometric strength across both the Chinese and English versions of the ULV-VFQ-50 underscores their value as a reliable tool for assessing visual functioning in individuals with ULV.^2^

## Conclusions

This study focused on a simplified version of 50 items selected from the comprehensive Chinese ULV-VFQ-150 to examine its psychometric functionality. The Chinese ULV-VFQ-50 demonstrates robust psychometric properties and is well suited for assessing visual function in individuals with ULV. Because of its simplicity and brevity, the ULV-VFQ-50 is poised to greatly facilitate clinical trials and multicenter and multilingual research endeavors.

## Supplementary Material

Supplement 1

Supplement 2

Supplement 3

Supplement 4

## References

[1] Van Der AAHPA, Comijs HC, Penninx BWJH, et al. Major depressive and anxiety disorders in visually impaired older adults. *Invest Ophthalmol Vis Sci*. 2015; 56: 849–854. 25604690 10.1167/iovs.14-15848

[2] Dagnelie G, Jeter PE, Adeyemo O. Optimizing the ULV-VFQ for clinical use through item set reduction: psychometric properties and trade-offs. *Transl Vis Sci Technol*. 2017; 6(3): 12. 10.1167/tvst.6.3.12 PMC5450924 28573076

[3] Adeyemo O, Jeter PE, Rozanski C, et al. Living with ultra-low vision: an inventory of self-reported visually guided activities by individuals with profound visual impairment. *Transl Vis Sci Technol*. 2017; 6(3): 10. 10.1167/tvst.6.3.10 PMC5450922 28573074

[4] Jeter PE, Rozanski C, Massof R, et al. Development of the ultra-low vision visual functioning questionnaire (ULV-VFQ). *Transl Vis Sci Technol*. 2017; 6(3): 11. 10.1167/tvst.6.3.11 PMC5450923 28573075

[5] Cong J, Wu X, Wang J, et al. Development of the chinese version of ultra-low vision visual functioning questionnaire-150. *Transl Vis Sci Technol*. 2023; 12(6): 9. 10.1167/tvst.12.6.9 PMC10275386 37310736

[6] Andrich D. A rating formulation for ordered response categories. *Psychometrika*. 1978; 43: 561–573.

[7] Luo G. A class of probabilistic unfolding models for polytomous responses. *J Math Psychol*. 2001; 45: 224–248. 11302711 10.1006/jmps.2000.1310

[8] Cantó-Cerdán M, Cacho-Martínez P, Lara-Lacárcel F, et al. Rasch analysis for development and reduction of symptom questionnaire for visual dysfunctions (SQVD). *Scientific Reports*. 2021; 11(1): 14855. 34290288 10.1038/s41598-021-94166-9 PMC8295373

[9] Pesudovs K, Burr JM, Harley C, et al. The development, assessment, and selection of questionnaires. *Optom Vis Sci*. 2007; 84(8): 663–674. 17700331 10.1097/OPX.0b013e318141fe75

[10] J.M L. Winsteps Rasch Tutorial. [(accessed on April 12, 2023)]; Available at: http://www.winsteps.com/a/winsteps-tutorial-4.pdf[J]. Accessed April 12, 2023.

[11] Summerfield AQ, Kitterick PT. Using Rasch analysis to assess and improve the measurement properties of a questionnaire with few items: the York Binaural Hearing-Related Quality of Life (YBHRQL) Questionnaire. *Ear and Hearing*. 2023; 44(6): 1526–1539. 37358331 10.1097/AUD.0000000000001400

[12] Linacre JM . A User's Guide to Winsteps/Ministeps Rasch-Model Computer Programs. Available at: https://www.winsteps.com/winman/copyright.htm. Accessed April 12, 2023.

[13] Wright B. Reasonable mean-square fit values. *Rasch Meas Trans*. 1994; 8: 370.

[14] Boone W J . Rasch analysis for instrument development: why, when, and how?. *CBE Life Sci Educ*. 2016; 15(4): rm4. 27856555 10.1187/cbe.16-04-0148 PMC5132390

[15] Linacre J M . Optimizing rating scale category effectiveness. *J Appl Measurement*. 2002; 3(1): 85–106. 11997586

[16] Jeter PE, Rozanski C, Massof R, et al. Development of the Ultra-Low Vision Visual Functioning Questionnaire (ULV-VFQ). *Transl Vis Sci Technol*. 2017; 6(3): 11. 10.1167/tvst.6.3.11 PMC5450923 28573075

[17] Armstrong RA . When to use the Bonferroni correction. *Ophthalmic Physiol Opt*. 2014; 34: 502–508. 24697967 10.1111/opo.12131

[18] Wan Y, Zhao L, Huang C, et al. Validation and comparison of the National Eye Institute Visual Functioning Questionnaire-25 (NEI VFQ-25) and the Visual Function Index-14 (VF-14) in patients with cataracts: a multicentre study. *Acta Ophthalmol*. 2021; 99(4): e480–e488. 32940410 10.1111/aos.14606 PMC8359188

[19] Khadka J, Gothwal VK, Mcalinden C, et al. The importance of rating scales in measuring patient-reported outcomes. *Health Qual Life Outcomes*. 2012; 10: 80. 22794788 10.1186/1477-7525-10-80 PMC3503574

[20] Bittner AK, Ibrahim MA, Haythornthwaite JA, et al. Vision test variability in retinitis pigmentosa and psychosocial factors. *Optom Vis Sci*. 2011; 88: 1496–1506. 21946786 10.1097/OPX.0b013e3182348d0b PMC3223543

