# Supplementary material for: Development and Psychometric Assessment of a Chinese Version of the Ultra-Low Vision Visual Functioning Questionnaire-50
Source: Transl Vis Sci Technol. 2024 Nov 18;13(11):20. doi: 10.1167/tvst.13.11.20 (PMC11578157; doi:10.1167/tvst.13.11.20)
Supplement: Supplement 1 [file tvst-13-11-20_s001.pdf]

ULV-VFQ 50

Respondent ID:

Instructions

This questionnaire consists of 50 items, each of which asks you to judge the difficulty of a performing a visual activity, with your current vision. Please answer all questions to the best of your ability.

The items deal with activities you may do on a day-to-day basis or have done in the past. Although you may complete many of these activities using auditory, tactile or a combination of sensory information including the use of a cane, a guide dog or help from another person, we would like you to focus on how difficult it is for you to complete the activities on your own using only your remaining vision. It is OK if the task is achieved using visual assistive devices, such as a magnifier or CCTV. Not every question may be applicable to your experience. Please answer as many questions as possible to the best of your ability.

Your answer choices are: “not applicable”, “not difficult”, “somewhat difficult”, “very difficult”, and “Impossible to see or do visually”.
Every question requires exactly one response.

If you are completing this questionnaire on-line and miss a question or have accidentally clicked on multiple responses, you will not be able to proceed to the next page of questions until the item is resolved. An error message will appear at the top of the question at issue. There are 5 questions per page for a total of 10 pages. You may not skip a question; however, you can take a break and come back to the questionnaire at any time. In order to save your responses and exit the survey, you must press "Next" or "Done" first (located at the bottom of each page), then "Exit Survey" (located in the upper right corner).

On the next page we will ask a few questions about your current vision that may help us understand your answers to the questionnaire items.

.

Copyright 2016 The Johns Hopkins University

Please answer the following 3 introductory questions about the history of your vision:

Was there ever a time when you were legally allowed to drive?

- » Yes
- » No
- » If yes, how long ago did you lose the ability to do so (in years):

Was there ever a time when you could recognize a face from 6 ft away?

- » Yes
- » No
- » If yes, how long ago did you lose the ability to do so (in years):

Do you still have any useable vision?

- » Yes
- » No
- » If no, how long ago did you lose the ability to do so (in years):

Please go to the next page to start answering the 50 questionnaire items.

1. How difficult is it for you to see a sunny spot in the garden to plant a tree or flower?

- ☐ » a not applicable
- ☐ » b not difficult
- ☐ » c somewhat difficult
- ☐ » d very difficult
- ☐ » e impossible to see or do visually

2. When standing outside on a cloudy day, how difficult is it for you to see the edge where a dark, green lawn meets the black pavement?

- ☐ » a not applicable
- ☐ » b not difficult
- ☐ » c somewhat difficult
- ☐ » d very difficult
- ☐ » e impossible to see or do visually

3. When watching an ice hockey game on a large TV, how difficult is it to see in which direction the players wearing dark uniforms are moving?

- ☐ » a not applicable
- ☐ » b not difficult
- ☐ » c somewhat difficult
- ☐ » d very difficult
- ☐ » e impossible to see or do visually

4. When signing a form, how difficult is it to visually locate the line you need to sign on?

- ☐ » a not applicable
- ☐ » b not difficult
- ☐ » c somewhat difficult
- ☐ » d very difficult
- ☐ » e impossible to see or do visually

5. How difficult is it to see a white tube of cream or lotion on a white bathroom counter?

- ☐ » a not applicable
- ☐ » b not difficult
- ☐ » c somewhat difficult
- ☐ » d very difficult
- ☐ » e impossible to see or do visually

6. How difficult is it for you to see your white plate on a dark tablecloth in a well-lit restaurant?

- ☐ » a not applicable
- ☐ » b not difficult
- ☐ » c somewhat difficult
- ☐ » d very difficult
- ☐ » e impossible to see or do visually

7. When facing a window on an overcast day in a coffee shop, how difficult is it for you to see pedestrians walking on the sidewalk?

- ☐ » a not applicable
- ☐ » b not difficult
- ☐ » c somewhat difficult
- ☐ » d very difficult
- ☐ » e impossible to see or do visually

8. How difficult is it to see if the dark red curtain is drawn in a brightly lit theater?

- ☐ » a not applicable
- ☐ » b not difficult
- ☐ » c somewhat difficult
- ☐ » d very difficult
- ☐ » e impossible to see or do visually

9. How difficult is to see if a mirror is clean in optimal lighting conditions?

- ☐ » a not applicable
- ☐ » b not difficult
- ☐ » c somewhat difficult
- ☐ » d very difficult
- ☐ » e impossible to see or do visually

10. How difficult is it to see a dark towel or washcloth hanging in the well-lit bathroom against a white wall?

- ☐ » a not applicable
- ☐ » b not difficult
- ☐ » c somewhat difficult
- ☐ » d very difficult
- ☐ » e impossible to see or do visually

11. How difficult is it for you to see a freshly-painted white crosswalk on dark pavement on a cloudy day?

- ☐ » a not applicable
- ☐ » b not difficult
- ☐ » c somewhat difficult
- ☐ » d very difficult
- ☐ » e impossible to see or do visually

12. When flying on a plane during the day, how difficult is it for you to determine if the window shade is open?

- ☐ » a not applicable
- ☐ » b not difficult
- ☐ » c somewhat difficult
- ☐ » d very difficult
- ☐ » e impossible to see or do visually

13. When sitting down at dinner, how difficult is it for you to see a large, red wine stain on your partner's white clothing?

- ☐ » a not applicable
- ☐ » b not difficult
- ☐ » c somewhat difficult
- ☐ » d very difficult
- ☐ » e impossible to see or do visually

14. When drawing a silhouette using a graphite pencil, how difficult is it to see the black pencil outline?

- ☐ » a not applicable
- ☐ » b not difficult
- ☐ » c somewhat difficult
- ☐ » d very difficult
- ☐ » e impossible to see or do visually

15. In a well-lit public restroom, how difficult is it to see a dark, gray sink on a white wall?

- ☐ » a not applicable
- ☐ » b not difficult
- ☐ » c somewhat difficult
- ☐ » d very difficult
- ☐ » e impossible to see or do visually

16. In a hotel room, how difficult is it for you to find the bathroom in the middle of the night if the bathroom light has been left on?

- ☐ » a not applicable
- ☐ » b not difficult
- ☐ » c somewhat difficult
- ☐ » d very difficult
- ☐ » e impossible to see or do visually

17. When attending a meeting, how difficult is it to locate the illuminated, white projection screen in a dark room?

- ☐ » a not applicable
- ☐ » b not difficult
- ☐ » c somewhat difficult
- ☐ » d very difficult
- ☐ » e impossible to see or do visually

18. At an airport, how difficult is it for you to recognize your brightly marked luggage on the baggage carousel?

- ☐ » a not applicable
- ☐ » b not difficult
- ☐ » c somewhat difficult
- ☐ » d very difficult
- ☐ » e impossible to see or do visually

19. How difficult is it to see if a server is standing at a nearby table 3 ft away so that you can get their attention?

- ☐ » a not applicable
- ☐ » b not difficult
- ☐ » c somewhat difficult
- ☐ » d very difficult
- ☐ » e impossible to see or do visually

20. When using a computer, how difficult is it for you to track a large, moving white cursor against a dark background?

- ☐ » a not applicable
- ☐ » b not difficult
- ☐ » c somewhat difficult
- ☐ » d very difficult
- ☐ » e impossible to see or do visually

21. How difficult is it for you to see the difference between a similarly sized or shaped orange bottle of laundry detergent and a white bottle of bleach?

- » a not applicable
- » b not difficult
- » c somewhat difficult
- » d very difficult
- » e impossible to see or do visually

22. How difficult is to see if your cell phone screen is on in a dark room?

- » a not applicable
- » b not difficult
- » c somewhat difficult
- » d very difficult
- » e impossible to see or do visually

23. How difficult is it for you to see if the horizontal or vertical blinds are open or closed on a sunny day?

- » a not applicable
- » b not difficult
- » c somewhat difficult
- » d very difficult
- » e impossible to see or do visually

24. In a well-lit public restroom, how difficult is it to see the dark paper towel dispenser or hand dryer on a white wall?

- » a not applicable
- » b not difficult
- » c somewhat difficult
- » d very difficult
- » e impossible to see or do visually

25. When shopping in the supermarket, how difficult is it to see the difference between a white bagel and pumpernickel bagel?

- » a not applicable
- » b not difficult
- » c somewhat difficult
- » d very difficult
- » e impossible to see or do visually

26. When attending a social function, how difficult is it to notice hand gestures when speaking to a fellow attendee standing 2 ft away?

- ☐ » a not applicable
- ☐ » b not difficult
- ☐ » c somewhat difficult
- ☐ » d very difficult
- ☐ » e impossible to see or do visually

27. How difficult is it for you to see the difference between white socks and black socks?

- ☐ » a not applicable
- ☐ » b not difficult
- ☐ » c somewhat difficult
- ☐ » d very difficult
- ☐ » e impossible to see or do visually

28. When taking a picture, how difficult is it to center the high contrast silhouette of a person on the digital display of the camera?

- ☐ » a not applicable
- ☐ » b not difficult
- ☐ » c somewhat difficult
- ☐ » d very difficult
- ☐ » e impossible to see or do visually

29. How difficult is it for you to see tables with white tablecloths against dark carpeting in a brightly lit restaurant?

- ☐ » a not applicable
- ☐ » b not difficult
- ☐ » c somewhat difficult
- ☐ » d very difficult
- ☐ » e impossible to see or do visually

30. When standing in the kitchen, how difficult is to see a clear empty glass on the counter?

- ☐ » a not applicable
- ☐ » b not difficult
- ☐ » c somewhat difficult
- ☐ » d very difficult
- ☐ » e impossible to see or do visually

31. When walking down a dimly lit hallway looking for a meeting room, how difficult is it to see if the lights are on in the room?

- ☐ » a not applicable
- ☐ » b not difficult
- ☐ » c somewhat difficult
- ☐ » d very difficult
- ☐ » e impossible to see or do visually

32. How difficult is it to see a white soccer ball rolling slowly down a black asphalt driveway?

- ☐ » a not applicable
- ☐ » b not difficult
- ☐ » c somewhat difficult
- ☐ » d very difficult
- ☐ » e impossible to see or do visually

33. How difficult is it for you to see white rice on a white plate?

- ☐ » a not applicable
- ☐ » b not difficult
- ☐ » c somewhat difficult
- ☐ » d very difficult
- ☐ » e impossible to see or do visually

34. How difficult is it for you to see if your computer monitor is on in a dark room?

- ☐ » a not applicable
- ☐ » b not difficult
- ☐ » c somewhat difficult
- ☐ » d very difficult
- ☐ » e impossible to see or do visually

35. When navigating an office building, how difficult is it to see if a glass door is open or closed?

- ☐ » a not applicable
- ☐ » b not difficult
- ☐ » c somewhat difficult
- ☐ » d very difficult
- ☐ » e impossible to see or do visually

36. When dressing a wound, how difficult is it to see a large blood stain on a white paper towel or gauze?

- ☐ » a not applicable
- ☐ » b not difficult
- ☐ » c somewhat difficult
- ☐ » d very difficult
- ☐ » e impossible to see or do visually

37. When painting or drawing, how difficult is it to select the appropriate shade of yellow from the color palette?

- ☐ » a not applicable
- ☐ » b not difficult
- ☐ » c somewhat difficult
- ☐ » d very difficult
- ☐ » e impossible to see or do visually

38. When setting a long table with white plates on a black tablecloth, how difficult is it to see whether the place settings are evenly spaced?

- ☐ » a not applicable
- ☐ » b not difficult
- ☐ » c somewhat difficult
- ☐ » d very difficult
- ☐ » e impossible to see or do visually

39. When walking through the woods on a sunny day, how difficult is it for you to see a deer from 30 yards away?

- ☐ » a not applicable
- ☐ » b not difficult
- ☐ » c somewhat difficult
- ☐ » d very difficult
- ☐ » e impossible to see or do visually

40. When standing at the gate in a bright airport, how difficult is it for you to see your gate number?

- ☐ » a not applicable
- ☐ » b not difficult
- ☐ » c somewhat difficult
- ☐ » d very difficult
- ☐ » e impossible to see or do visually

41. When standing at a white countertop, how difficult is it for you to notice a dark pill lying right in front of you?

- [ ] » a not applicable
- [ ] » b not difficult
- [ ] » c somewhat difficult
- [ ] » d very difficult
- [ ] » e impossible to see or do visually

42. When riding in a car at night, how difficult is it to see oncoming headlights?

- [ ] » a not applicable
- [ ] » b not difficult
- [ ] » c somewhat difficult
- [ ] » d very difficult
- [ ] » e impossible to see or do visually

43. How difficult to see if a candle is lit in a dark room?

- [ ] » a not applicable
- [ ] » b not difficult
- [ ] » c somewhat difficult
- [ ] » d very difficult
- [ ] » e impossible to see or do visually

44. When standing at a black countertop, how difficult is it for you to notice a white pill lying right in front of you?

- [ ] » a not applicable
- [ ] » b not difficult
- [ ] » c somewhat difficult
- [ ] » d very difficult
- [ ] » e impossible to see or do visually

45. How difficult is it for you to identify if the lights are on in the room?

- [ ] » a not applicable
- [ ] » b not difficult
- [ ] » c somewhat difficult
- [ ] » d very difficult
- [ ] » e impossible to see or do visually

46. How difficult is it for you to locate a window in a dark room on a sunny day?

- » a not applicable
- » b not difficult
- » c somewhat difficult
- » d very difficult
- » e impossible to see or do visually

47. When standing outside at high noon, how difficult is it for you to tell whether it is sunny outside?

- » a not applicable
- » b not difficult
- » c somewhat difficult
- » d very difficult
- » e impossible to see or do visually

48. When camping on a moonless, cloudy night, how difficult is it to see if the flashlight you are holding is on?

- » a not applicable
- » b not difficult
- » c somewhat difficult
- » d very difficult
- » e impossible to see or do visually

49. When camping on a moonless, cloudy night, how difficult is it to see the large burning campfire from 5ft away?

- » a not applicable
- » b not difficult
- » c somewhat difficult
- » d very difficult
- » e impossible to see or do visually

50. How difficult is it to see a person's black tie against their white shirt from 2 ft away?

- » a not applicable
- » b not difficult
- » c somewhat difficult
- » d very difficult
- » e impossible to see or do visually

Thank you very much for completing the questionnaire!
