# Supplementary material for: Development and Psychometric Assessment of a Chinese Version of the Ultra-Low Vision Visual Functioning Questionnaire-50
Source: Transl Vis Sci Technol. 2024 Nov 18;13(11):20. doi: 10.1167/tvst.13.11.20 (PMC11578157; doi:10.1167/tvst.13.11.20)
Supplement: Supplement 2 [file tvst-13-11-20_s002.pdf]

ULV-VFQ 50

调查对象 ID：

说明：

该问卷包括 50 题，每题都要求您根据当前视力情况判断进行视觉活动的难度，请您尽可能回答所有问题。
这些题目涉及您可能每天或过去进行过的活动。尽管您可以使用听觉、触觉或感觉信息组合完成其中许多活动，包括利用手杖、导盲犬或他人的帮助，我们希望您专注于仅使用您的视力评判自行完成活动有多困难。可以使用视觉辅助设备（如放大镜或显示器）完成任务。并非每个问题都适用于您的经历。请您尽可能多的回答问题。

您的答案选择有："不适用"、"不困难"、"有些困难"、"非常困难"、"因为视力问题无法完成/无法看到"。
每个问题只需要一个答案。

如果您在线填写此调查问卷而遗漏了一个问题或无意点击了多个回答，您将无法进入下一页的问题，直到问题得到解决。报错消息将显示在问题的顶部。每页有 5 个问题，共 30 页。您不能跳过问题，但是您可以休息一下，并随时返回继续答卷。为了保存您的回答和退出问卷，您必须先按"下一步"或"完成"（位于每页底部），然后按"退出调查"（位于右上角）。

在下一页，我们将询问关于您当前视力的几个问题，这可能有助于我们理解您对问卷项目的回答。

请回答以下3个关于您的视力历史的介绍性问题：

1、 您是否曾获取驾照？
是
否

-如果是，您是在多久之前失去了这样的资格？（以年为单位）

2、您此前是否曾经可以从 6 英尺距离外辨认出一张脸？
是
否

-如果是，您在多久之前失去了这样的能力？（以年为单位）

3、您是否还有可用的视力？

有
否

-如果没有，您在多久前失去了这样的视力？（以年为单位）：

请至下一页开始回答 50 个问卷题目

1. 当收到一张打印页时，看出哪一面是空白的、哪一面是打印的有多困难？

☐»a 不适用

☐»b 不难

☐»c 有些困难

☐»d 非常困难

☐»e 因为视力问题无法完成/无法看到

2. 对您来说，在花园里找到一个可以种树或种花的阳光充足的地方有多困难？

☐»a 不适用

☐»b 不难

☐»c 有些困难

☐»d 非常困难

☐»e 因为视力问题无法完成/无法看到

3. 一位家庭成员或朋友站在5英尺远的地方，要根据其身高或体格来认出他/她，对您来说

有多困难?

☐»a 不适用

☐»b 不难

☐»c 有些困难

☐»d 非常困难

☐»e 因为视力问题无法完成/无法看到

4. 当在光线充足的厨房中清洁白色台面时，看到一大团葡萄果冻污渍对您来说有多困难？

☐»a 不适用

☐»b 不难

☐»c 有些困难

☐»d 非常困难

☐»e 因为视力问题无法完成/无法看到

5. 在您签署表格时，找到您需要签署名字的位置（有下划线的地方）？

☐»a 不适用

☐»b 不难

☐»c 有些困难

☐»d 非常困难

☐»e 因为视力问题无法完成/无法看到

6. 您在日光灯下，区分一条卡其裤和一条深色牛仔裤有多困难？

☐»a 不适用

☐»b 不难

☐»c 有些困难

☐»d 非常困难

☐»e 因为视力问题无法完成/无法看到

7. 下国际象棋时，在白色方格上看到黑色的棋子有多困难？

☐»a 不适用

☐»b 不难

☐»c 有些困难

☐»d 非常困难

☐»e 因为视力问题无法完成/无法看到

8. 当看护一个幼儿时，注视他/她在房间里走动有多困难？

☐»a 不适用

☐»b 不难

☐»c 有些困难

☐»d 非常困难

☐»e 因为视力问题无法完成/无法看到

9. 在光线明亮的餐厅，看到深色桌布上您的白色盘子有多困难？

☐»a 不适用

☐»b 不难

☐»c 有些困难

☐»d 非常困难

☐»e 因为视力问题无法完成/无法看到

10. 在最佳光照条件下，观察镜子是否洁净有多困难？

☐»a 不适用

□»b 不难

□»c 有些困难

□»d 非常困难

□»e 因为视力问题无法完成/无法看到

11. 在多云的天气里，您分辨深色路面上新刷的白色斑马线有多困难？

□»a 不适用

□»b 不难

□»c 有些困难

□»d 非常困难

□»e 因为视力问题无法完成/无法看到

12. 当使用辅助设备(如放大镜或显示器)，在黑色的屏幕上看到白色或黄色的大号字符，有多困难?

□»a 不适用

□»b 不难

□»c 有些困难

□»d 非常困难

□»e 因为视力问题无法完成/无法看到

13. 您看出白色杯子中有多少咖啡或红茶有多困难？

□»a 不适用

□»b 不难

□»c 有些困难

□»d 非常困难

□»e 因为视力问题无法完成/无法看到

14. 在不熟悉的商店购物时，仅使用您现有的视力找到收银台有多困难？

□»a 不适用

□»b 不难

□»c 有些困难

□»d 非常困难

☐ »e 因为视力问题无法完成/无法看到

15. 烘焙时，看到白色量杯里的深色红糖有多困难？

☐»a 不适用

☐»b 不难

☐»c 有些困难

☐»d 非常困难

☐»e 因为视力问题无法完成/无法看到

16. 在接待处，看到一个穿深色衣服的客人站在白色墙壁前有多困难？

☐»a 不适用

☐»b 不难

☐»c 有些困难

☐»d 非常困难

☐»e 因为视力问题无法完成/无法看到

17. 当您使用您现有的视力，看到台式计算器上黑色按钮上的白色数字有多困难？

☐»a 不适用

☐»b 不难

☐»c 有些困难

☐»d 非常困难

☐»e 因为视力问题无法完成/无法看到

18. 当您在机场时，从行李转盘上找到您做过明显标记的行李，有多困难？

☐»a 不适用

☐»b 不难

☐»c 有些困难

☐»d 非常困难

☐»e 因为视力问题无法完成/无法看到

19. 当使用您的辅助设备时，您看到白色日历上写的黑色粗体标记有多困难？

☐»a 不适用

☐»b 不难

☐»c 有些困难

☐»d 非常困难

☐»e 因为视力问题无法完成/无法看到

20. 在光线充足的公共卫生间，看到白色墙壁前的白色马桶，有多困难？

☐»a 不适用

☐»b 不难

☐»c 有些困难

☐»d 非常困难

☐»e 因为视力问题无法完成/无法看到

21. 喂孩子的时候，看到小孩张开的嘴巴有多困难？

☐»a 不适用

☐»b 不难

☐»c 有些困难

☐»d 非常困难

☐»e 因为视力问题无法完成/无法看到

22. 在黑暗的房间里，您察觉自己手机屏幕是否亮着有多困难？

☐»a 不适用

☐»b 不难

☐»c 有些困难

☐»d 非常困难

☐»e 因为视力问题无法完成/无法看到

23. 看到一根绿藤上的一个红番茄有多困难？

☐»a 不适用

☐»b 不难

☐»c 有些困难

☐»d 非常困难

☐»e 因为视力问题无法完成/无法看到

24. 您辨别白袜子和黑袜子，有多困难？

☐»a 不适用

☐»b 不难

☐»c 有些困难

☐»d 非常困难

☐»e 因为视力问题无法完成/无法看到

25. 当您站在厨房时，看到厨房台面上一个空的干净玻璃杯有多困难？

☐»a 不适用

☐»b 不难

☐»c 有些困难

☐»d 非常困难

☐»e 因为视力问题无法完成/无法看到

26. 在黑色沥青路面上看到白色的足球缓缓滚动有多困难？

☐»a 不适用

☐»b 不难

☐»c 有些困难

☐»d 非常困难

☐»e 因为视力问题无法完成/无法看到

27. 当您走在一个室内购物中心里时，看到距离您5英尺（1.5米）外的自动扶梯口有多困难？

☐»a 不适用

☐»b 不难

☐»c 有些困难

☐»d 非常困难

☐»e 因为视力问题无法完成/无法看到

28. 当您站在办公室门口，在2英尺（0.6米）远的地方看到3英寸大小的房间号码有多困难？

☐»a 不适用

☐»b 不难

☐»c 有些困难

☐»d 非常困难

☐»e 因为视力问题无法完成/无法看到

29. 在白色盘子上看到白色米粒有多困难？

☐»a 不适用

☐»b 不难

☐»c 有些困难

☐»d 非常困难

☐»e 因为视力问题无法完成/无法看到

30. 在黑暗的房间里，您察觉您的电脑显示器是否亮着，有多困难？

☐»a 不适用

☐»b 不难

☐»c 有些困难

☐»d 非常困难

☐»e 因为视力问题无法完成/无法看到

31. 在一个光线充足的公共卫生间里，看到出口在哪里有多困难？

☐»a 不适用

☐»b 不难

☐»c 有些困难

☐»d 非常困难

☐»e 因为视力问题无法完成/无法看到

32. 在办公楼里走动时，看到玻璃门是打开还是关闭的有多困难？

☐»a 不适用

☐»b 不难

☐»c 有些困难

☐»d 非常困难

☐»e 因为视力问题无法完成/无法看到

33. 在黑色电话上辨认大的白色数字号码有多困难？

☐»a 不适用

☐»b 不难

☐»c 有些困难

☐»d 非常困难

☐»e 因为视力问题无法完成/无法看到

34. 在光照充足的卫生间，您给自己梳理头发有多困难？

☐»a 不适用

☐»b 不难

☐»c 有些困难

☐»d 非常困难

☐»e 因为视力问题无法完成/无法看到

35. 在黑暗的房间里，您看清数字闹钟上看的大号数字有多困难？

☐»a 不适用

☐»b 不难

☐»c 有些困难

☐»d 非常困难

☐»e 因为视力问题无法完成/无法看到

36. 当您在阳光明媚的日子穿过公园时，看到一只10米开外的流浪狗有多困难？

☐»a 不适用

☐»b 不难

☐»c 有些困难

☐»d 非常困难

☐»e 因为视力问题无法完成/无法看到

37. 分辨《人民日报》和《文汇报》的不同，有多困难？

☐»a 不适用

☐»b 不难

☐»c 有些困难

☐»d 非常困难

☐»e 因为视力问题无法完成/无法看到

38. 用放大镜查找写在您电话簿上的电话号码有多困难？

☐»a 不适用

☐»b 不难

☐»c 有些困难

☐»d 非常困难

☐»e 因为视力问题无法完成/无法看到

39. 参加会议或上课时，要想看您的常坐的位置是否空着有多困难？

☐»a 不适用

☐»b 不难

☐»c 有些困难

☐»d 非常困难

☐»e 因为视力问题无法完成/无法看到

40. 当您拿着大号字的纸牌时，要辨别出您拿着带人像的扑克牌有多困难？

☐»a 不适用

☐»b 不难

☐»c 有些困难

☐»d 非常困难

☐»e 因为视力问题无法完成/无法看到

41. 在烹饪牛排时，用眼睛去判断其熟度或颜色变深的程度有多困难？

☐»a 不适用

☐»b 不难

☐»c 有些困难

☐»d 非常困难

☐»e 因为视力问题无法完成/无法看到

42. 在光线明亮的机场，站在登机口，看到登机口号有多困难？

☐»a 不适用

☐»b 不难

☐»c 有些困难

☐»d 非常困难

☐»e 因为视力问题无法完成/无法看到

43. 白天，看到暴风雨临近时的乌云有多困难？

☐»a 不适用

☐»b 不难

☐»c 有些困难

☐»d 非常困难

☐»e 因为视力问题无法完成/无法看到

44. 在医生办公室，在光线充足的候诊室里看到一个空位有多困难？

☐»a 不适用

☐»b 不难

☐»c 有些困难

☐»d 非常困难

☐»e 因为视力问题无法完成/无法看到

45. 当站在白色柜台前时，您看到您正前方柜台上放着的深色药丸有多困难？

☐»a 不适用

☐»b 不难

☐»c 有些困难

☐»d 非常困难

☐»e 因为视力问题无法完成/无法看到

46. 在光线明亮的车库中走动而不撞到障碍物有多困难？

☐»a 不适用

☐»b 不难

☐»c 有些困难

☐»d 非常困难

☐»e 因为视力问题无法完成/无法看到

47. 晚上您坐车时，看到迎面而来的汽车大灯有多困难？

☐»a 不适用

☐»b 不难

☐»c 有些困难

☐»d 非常困难

☐»e 因为视力问题无法完成/无法看到

48. 在黑暗的房间里，看到蜡烛是否是点燃的有多困难？

☐»a 不适用

☐»b 不难

☐»c 有些困难

☐»d 非常困难

☐»e 因为视力问题无法完成/无法看到

49. 辨认房间里的灯是否亮着，有多困难？

☐»a 不适用

☐»b 不难

☐»c 有些困难

☐»d 非常困难

☐»e 因为视力问题无法完成/无法看到

50. 当您正午站在室外时，您判断此际是否是晴天有多困难？

☐»a 不适用

☐»b 不难

☐»c 有些困难

☐»d 非常困难

☐»e 因为视力问题无法完成/无法看到

非常感谢您完成本次问卷！
