# Supplementary figures and images for: Development and Psychometric Assessment of a Chinese Version of the Ultra-Low Vision Visual Functioning Questionnaire-50
Source: Transl Vis Sci Technol. 2024 Nov 18;13(11):20. doi: 10.1167/tvst.13.11.20 (PMC11578157; doi:10.1167/tvst.13.11.20)

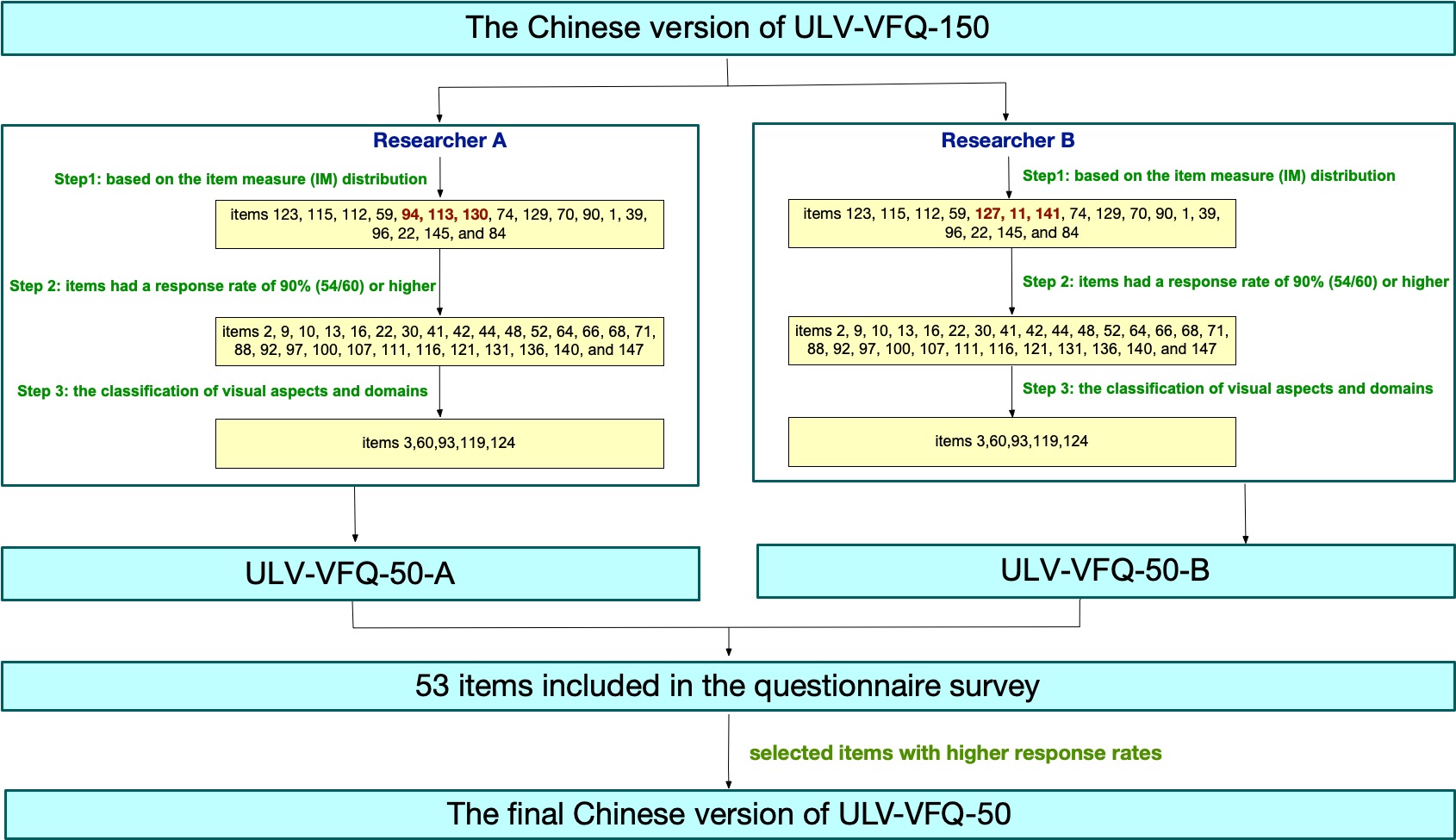

Supplement: Supplement 3 [file tvst-13-11-20_s003.jpg]
