# Supplementary material for: Development and Psychometric Assessment of a Chinese Version of the Ultra-Low Vision Visual Functioning Questionnaire-50
Source: Transl Vis Sci Technol. 2024 Nov 18;13(11):20. doi: 10.1167/tvst.13.11.20 (PMC11578157; doi:10.1167/tvst.13.11.20)
Supplement: Supplement 4 [file tvst-13-11-20_s004.pdf]

(Supplementary Materials 4) Table 1s: ZSTD of Contrast-related Items

Items	Infit ZSTD	Visual Aspects
1	2.64	Contrast (CO)
9	-0.75	Contrast (CO)
10	0.3	Contrast (CO)
11	-0.85	Contrast (CO)
13	1.09	Contrast (CO)
22	-0.79	Contrast (CO)
39	-1.73	Contrast (CO)
41	-1.23	Contrast (CO)
42	0.44	Contrast (CO)
48	1.7	Contrast (CO)
52	-0.88	Contrast (CO)
59	-1.79	Contrast (CO)
64	-0.9	Contrast (CO)
66	-5.08	Contrast (CO)
70	5.92	Contrast (CO)
71	0.84	Contrast (CO)
74	-3.33	Contrast (CO)
84	0.22	Contrast (CO)
88	-5.98	Contrast (CO)
92	-4.05	Contrast (CO)
100	-4.23	Contrast (CO)
107	-4.91	Contrast (CO)
116	-0.97	Contrast (CO)
123	0.6	Contrast (CO)
127	-0.42	Contrast (CO)
128	-1.25	Contrast (CO)
141	-1.63	Contrast (CO)

As shown in Table 1s, during the development of the ULV-VFQ-50, 27 contrast-related items from the full ULV-VFQ-150 were retained. Although the retained items generally exhibited favorable fit, specific contrast items, such as Item 70, showed poorer fit statistics (Infit ZSTD = 5.92), suggesting they were less aligned with the overall measurement model. In contrast, other retained contrast items performed within acceptable fit ranges, contributing to the robustness and reliability of the 50-item version.
